# Elucidating *N*-acyl amino acids as a model protoamphiphilic system

**DOI:** 10.1038/s42004-022-00762-9

**Published:** 2022-11-09

**Authors:** Manesh Prakash Joshi, Ashwin Uday, Sudha Rajamani

**Affiliations:** Department of Biology, Indian Institute of Science Education and Research Pune, Pune, 411008 India

**Keywords:** Origin of life, Lipids

## Abstract

Protoamphiphiles are prebiotically-plausible moieties that would have constituted protocell membranes on early Earth. Although prebiotic soup would have contained a diverse set of amphiphiles capable of generating protocell membranes, earlier studies were mainly limited to fatty acid-based systems. Herein, we characterize N-acyl amino acids (NAAs) as a model protoamphiphilic system. To the best of our knowledge, we report a new abiotic route in this study for their synthesis under wet-dry cycles from amino acids and monoglycerides via an ester-amide exchange process. We also demonstrate how N-oleoyl glycine (NOG, a representative NAA) results in vesicle formation over a broad pH range when blended with a monoglyceride or a fatty acid. Notably, NOG also acts as a substrate for peptide synthesis under wet-dry cycles, generating different lipopeptides. Overall, our study establishes NAAs as a promising protoamphiphilic system, and highlights their significance in generating robust and functional protocell membranes on primitive Earth.

## Abstract (web summary)

Protoamphiphiles – prebiotically plausible components of protocell membranes – could originate from a diverse set of amphiphiles, but studies so far have mostly been limited to fatty acid-based model systems. Here, the authors elucidate the abiotic synthesis of N-acyl amino acids under wet-dry cycles from amino acids and monoglycerides, characterize their vesicle formation and ability to act as a substrate for lipopeptide synthesis, thereby demonstrating N-acyl amino acids as a promising model protoamphiphile.

## Introduction

Compartmentalization is one of the key features of cellular life and would have also been central to its emergence on early Earth^1,2^. Before the advent of lipid-producing enzymatic machinery, early protocell membranes would have been generated from amphiphiles in the surrounding environment. Such prebiotic amphiphiles serving as membrane components of early protocells are called protoamphiphiles^3^. In this context, single-chain amphiphiles (SCAs) are promising protoamphiphile candidates, given their ability to form membranes^4^ and potential availability on early Earth^5,6^. Although many SCAs can be envisioned as protoamphiphiles, the focus has mainly remained on fatty acids as a model protoamphiphilic system^7–12^. This excessive dependence on fatty acids overlooks other interesting properties provided by several other putative protoamphiphiles to protocell membranes that could have implications for their sustenance and functioning. Therefore, it is crucial to systematically identify and characterize other SCAs that deem fit to be protoamphiphiles, to better understand the origin and evolution of protocell membranes.

*N*-acyl amino acids (NAAs) constitute an intriguing class of SCAs containing covalently-linked fatty acid and amino acid moieties. This amalgamation of two prebiotically important molecules within a single chemical species might have enabled NAAs to exploit the properties of both these molecules while also acquiring new emergent properties. Incidentally, NAAs are involved in several biological processes in extant biology^13^, having widespread commercial applications as antimicrobial agents^14^ and drug delivery systems^15^. In a prebiotic context, NAAs have been investigated for their ability to assist fatty acid-based membranes in vesicle growth and RNA localization on membrane surfaces^16,17^. However, NAAs themselves remain unexplored as a model protoamphiphilic system. We recently demonstrated NAA formation in a phospholipid-amino acid system under prebiotically-pertinent wet-dry cycling conditions and showed their vesicle-forming properties at acidic pH^3^. Herein, we report significant advancements in our systematic characterization of NAAs as a model protoamphiphilic system. To the best of our knowledge, we demonstrate a novel, prebiotically plausible route for their synthesis; their vesicle-forming ability over a broad pH range when blended with other amphiphiles; and their potential to act as a substrate for peptide synthesis, which generates different lipopeptides. These results elucidate the potential of NAAs to act as a model protoamphiphilic system that could have generated stable and functional membrane compartments during the origin and early evolution of life.

## Results and discussion

### Synthesis of NAAs from a monoglyceride and an amino acid under wet-dry cycles

**Fig. 1:**
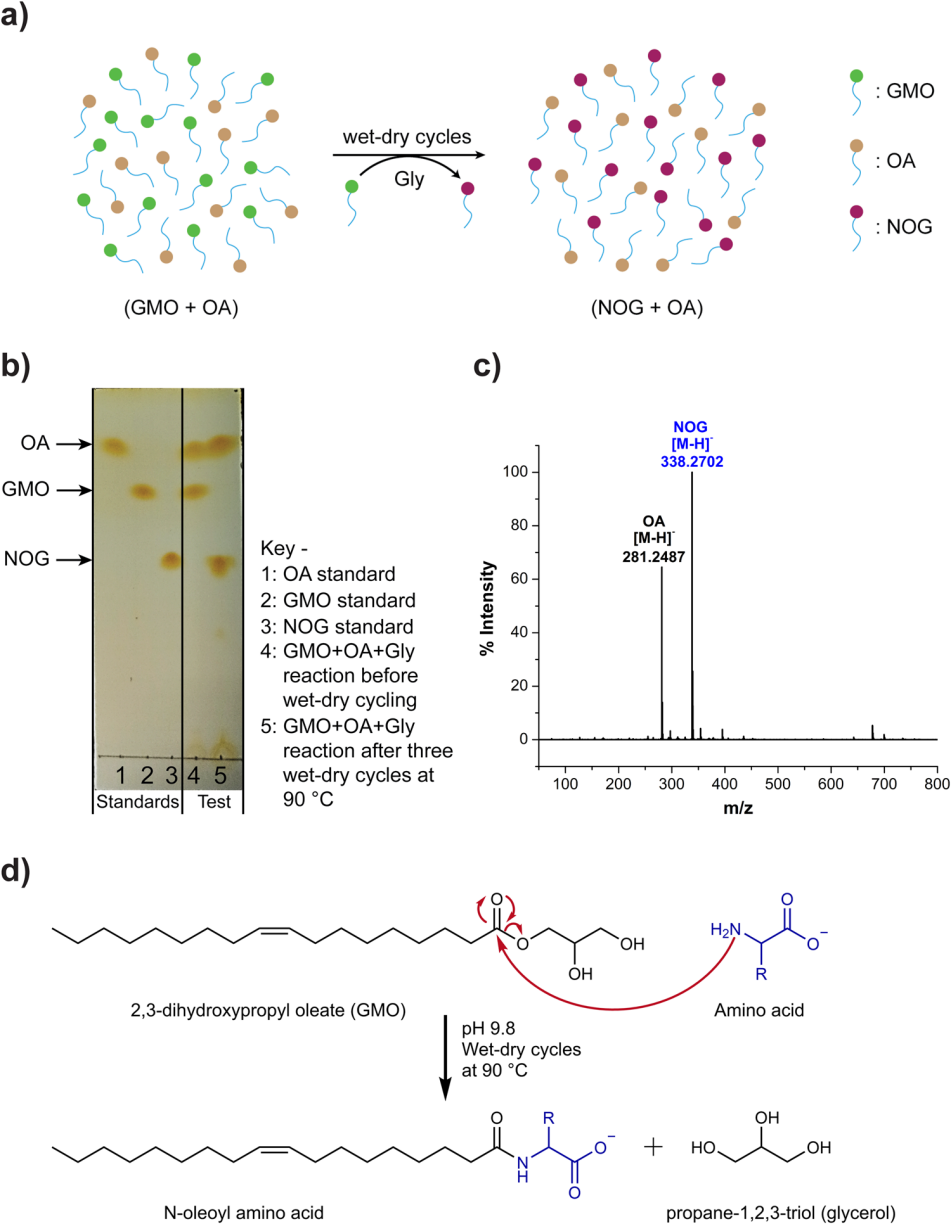
Conversion of glycerol 1-monooleate (GMO) to *N*-oleoyl glycine (NOG) in the presence of glycine (Gly) under three wet-dry cycles at 90 °C. The starting concentrations of GMO and Gly were 3 mM and 200 mM, respectively. Initial pH of the reaction was 9.8. The duration of each wet-dry cycle was 24 h. **a** Schematic overview of the reaction, **b** TLC analysis shows the complete disappearance of the GMO spot with a concurrent appearance of the NOG spot after wet-dry cycling (lane 5), **c** HRMS analysis (negative mode) of the final lipid content shows a prominent peak corresponding to NOG (calculated: 338.2701; observed: 338.2702; mass error = 0.3 ppm). Another peak at 281 corresponds to oleic acid (OA) (calculated: 281.2486; observed: 281.2487; mass error = 0.3 ppm), which was added in the initial reaction to increase the solubility of GMO, **d** A plausible mechanism for NAA synthesis from GMO and amino acid through ester-amide exchange reaction.

The NAA synthesis that we reported earlier occurs via an ester-amide exchange reaction between phospholipids and amino acids^3^. However, phospholipids are structurally-complex lipids, which themselves depend on SCA precursors for their synthesis in biosynthetic^18^ as well as prebiotically plausible enzyme-free reactions^19–22^. Therefore, we tested whether chemically-simpler, ester linkage-containing SCAs like monoglycerides can act as a precursor for NAA synthesis under prebiotically-relevant conditions. We used glycerol 1-monooleate (GMO) (Supplementary Fig. 1) as a representative monoglyceride, where a mixture of GMO and oleic acid (OA) (3 mM each) was subjected to three wet-dry cycles each of 24-h duration at 90 °C, in the presence of 200 mM glycine (Gly) at pH 9.8 (Fig. 1a). OA was added to increase the overall solubility of the GMO in water, where OA increases its solubility via vesicle formation (Supplementary Fig. 2). The formation of *N*-oleoyl glycine (NOG; a Gly-containing NAA) was evaluated by thin-layer chromatography (TLC). Upon TLC analysis, we observed the complete disappearance of the GMO spot with a concurrent appearance of the NOG spot, indicating the conversion of GMO to NOG (Fig. 1b). Notably, this NOG spot did not appear in other control reactions (Supplementary Figs. 3 and 4). NOG formation was further confirmed by analyzing the final lipid content of the reaction using high-resolution mass spectrometry (HRMS), which showed a prominent peak corresponding to NOG with high accuracy (Fig. 1c). These results were also corroborated by ^1^H-NMR analysis of the lipid content (Supplementary Fig. 5). Pertinently, we also observed the formation of other amino acid-containing NAAs, when Gly in the reaction was replaced with alanine, lysine, serine, and valine (Supplementary Figs. 6–9).

The conversion of GMO to NAA in the presence of an amino acid likely follows an ester-amide exchange mechanism (Fig. 1d), similar to the one we reported earlier for the phospholipid-amino acid reaction^3^. At alkaline pH, the nucleophilic amino group of an amino acid attacks the carbonyl carbon of the ester linkage in GMO, thereby generating a corresponding NAA via amide bond formation. To our knowledge, this describes a new, prebiotically-pertinent route for NAA synthesis, which supports their potential availability on early Earth. Importantly, it also highlights the significance of ester-amide exchange reaction in the prebiotic synthesis of protoamphiphiles, in addition to protopeptide synthesis^23^. Plausibly, at some stage during the evolution of life, the energy source required to drive the thermodynamically unfavorable synthesis of biologically-relevant molecules would have been shifted from environmental conditions (temperature, pH etc.) to chemical activation. In this regard, the NAAs synthesis has also been shown to occur via activation chemistry^16,17^, suggesting their plausible presence during the different stages of life’s evolution.

### Vesicle formation by NAA-based mixed amphiphile systems over a wide range of pH

**Fig. 2:**
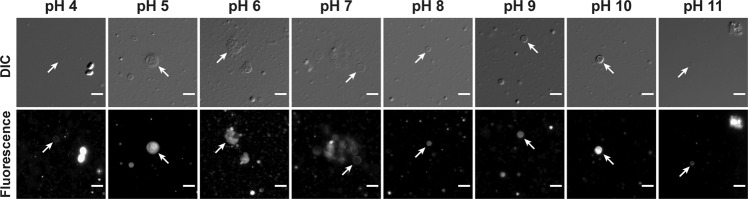
Vesicle formation behavior of the NOG + GMO mixed system. A mixture of NOG and GMO (6 mM total amphiphile; 2:1 ratio) generates vesicles from pH 4 to 11, as observed under differential interference contrast (DIC) and fluorescence microscopy. Vesicles are indicated by white arrows. For fluorescence-based imaging, vesicles were stained with 10 μM of an amphiphilic dye named Octadecyl Rhodamine B Chloride (R18). The scale bar is 10 µm.

Subsequently, we explored the relevance of NAAs as a model protocell membrane using NOG as a representative. Most conventional fatty acid-based systems are unable to form vesicles at acidic pH, which limits their ability to generate protocell compartments under a diverse pH regime. Earlier, we showed that NOG forms vesicles in the acidic range from pH 5 to 6, indicating that its apparent pK_a_ lies in this range^3^. Given that monoglycerides facilitate SCA vesicle formation towards the alkaline pH from their apparent pK_a_, we asked whether a composite mixture of NOG and GMO could form vesicles over a broad pH range. Indeed, the NOG + GMO mixed system (6 mM total amphiphile; 2:1 ratio) generated vesicles throughout from pH 4 to 11 (Fig. 2 and Supplementary Figs. 10–17). The hydroxyl groups in the glycerol part of GMO might stabilize the deprotonated NOG species via hydrogen bonding, allowing them to form vesicles over this broad pH range. However, the overall vesicle-forming propensity, as well as the size, shape, and lamellarity of the resultant vesicles seem to vary across different pH values. Nevertheless, it demonstrates that monoglycerides can facilitate the membrane assembly of NAAs over a diverse pH range in addition to acting as a precursor for their synthesis (Fig. 1).

**Fig. 3:**
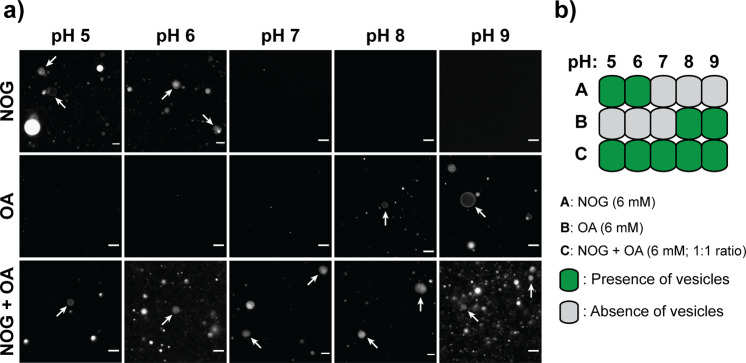
Vesicle formation behavior of the NOG + OA mixed system. **a** Fluorescence microscopy shows that 6 mM NOG and 6 mM OA by themselves generate vesicles at pH 5, 6, and pH 8, 9, respectively. However, the NOG + OA mixed system (6 mM total amphiphile; 1:1 ratio) forms vesicles across pH 5 to 9. Vesicles are indicated by white arrows. For fluorescence-based imaging, vesicles were stained with 10 μM of an amphiphilic dye named Octadecyl Rhodamine B Chloride (R18). The scale bar is 10 µm, **b** Schematic representation of the microscopy results that were shown in **a**.

Interestingly, the conversion of GMO to NOG in our reaction eventually led to a new heterogeneous amphiphile system containing NOG and OA (Fig. 1a). It is an intriguing mixed system as both its components have their own optimum pH range of vesicle formation (NOG: pH 5–6, OA: pH 8–9). Also, being present as co-solutes in the prebiotic soup, NAAs and fatty acids would likely have affected each other’s self-assembly behavior. Therefore, we evaluated the vesicle formation behavior of the NOG + OA mixed system (6 mM total amphiphile; 1:1 ratio) at different pH values. Consistent with earlier studies^3,24^, NOG (6 mM) formed vesicles at pH 5 and 6, whereas OA (6 mM) did so at pH 8 and 9. However, the NOG + OA mixed system generated vesicles across pH 5 to 9 (Fig. 3 and Supplementary Figure 18), particularly even at the physiological pH range around 7, where both its components were unable to form vesicles by themselves. At pH 7, the efficient hydrogen bonding between predominantly-present deprotonated NOG and protonated OA species can generate pseudodiacyl structures that favor bilayer formation. Primarily, this demonstrates how amphiphiles affect each other’s physicochemical properties when in a mixture, which could also result in new emergent properties, like vesicle formation at pH 7 in this case. Notably, these results might have implications in understanding NAAs role in biological cells, and towards developing NAA-based drug delivery systems, both of which operate at physiological pH. Altogether, these results highlight NOG’s ability to generate robust protocell membranes over a diverse pH range when mixed with amphiphiles like GMO or OA.

### NOG acts as a substrate for peptide chain growth under wet-dry cycles to generate lipopeptides

**Fig. 4:**
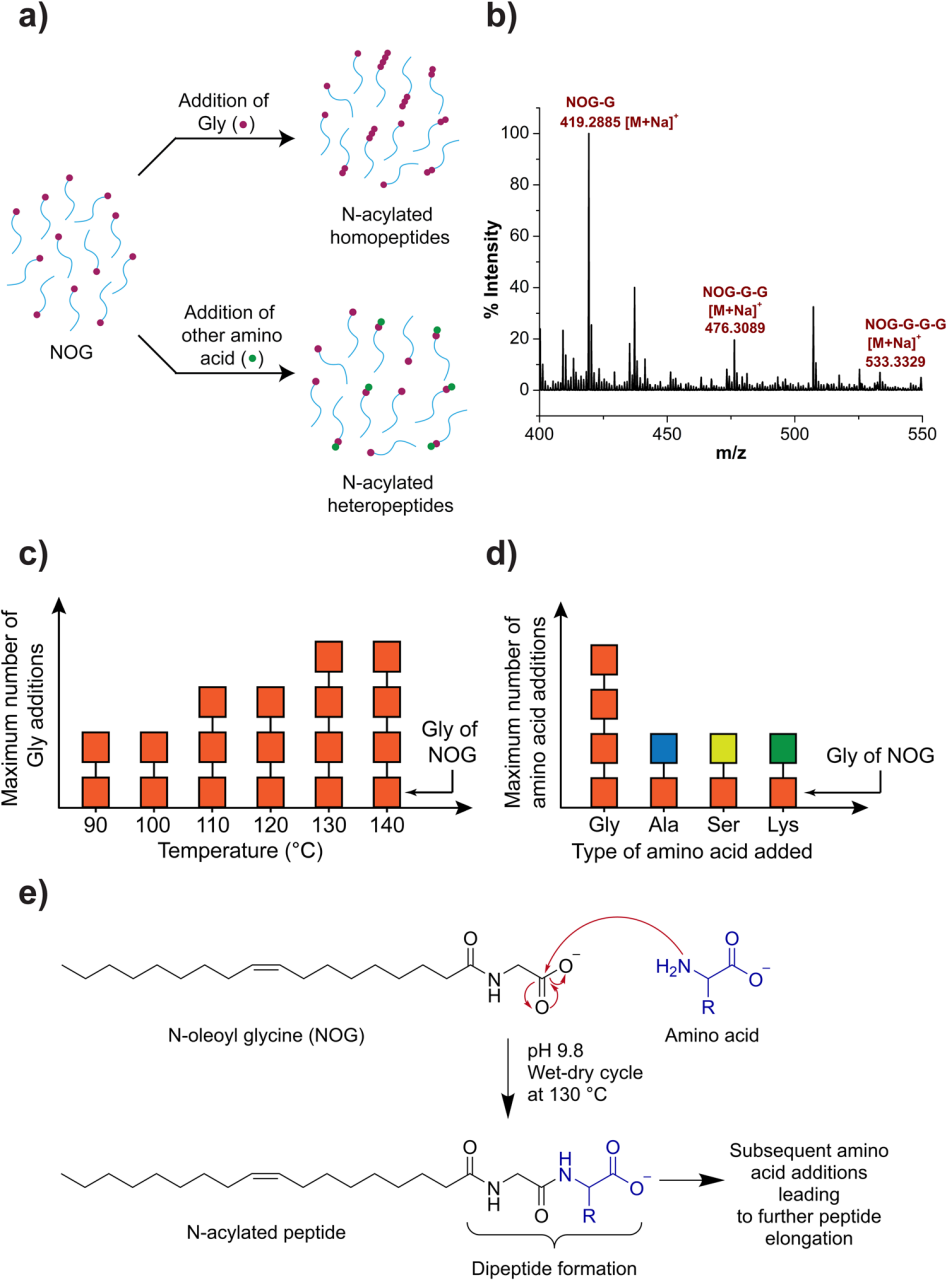
The addition of amino acids to the NOG head group under wet-dry cycles results in the synthesis of *N*-acylated peptides. The starting concentrations of NOG and Gly were 3 mM and 200 mM, respectively. Initial reaction pH was 9.8. Reaction was subjected to a single wet-dry cycle of 24 h at 130 °C. **a** Schematic overview of the reaction, **b** HRMS analysis (positive mode) of the final lipid content of the NOG + Gly reaction shows up to three Gly additions to the NOG head group (full spectrum shown in Supplementary Fig. 19), **c** The maximum number of Gly additions increases with increasing temperature, **d** Single amino acid addition is observed for the reaction of NOG with alanine, serine, and lysine performed under similar reaction conditions as that of the NOG + Gly reaction (see Supplementary Figs. 19–27 for the original HRMS data for **c** and **d**), **e** A plausible mechanism of the reaction, where the acid-amine coupling between NOG and amino acid results in the formation of *N*-acylated peptides.

Another potential application of NAAs is their ability to act as a substrate for peptide synthesis under prebiotically pertinent wet-dry cycling conditions, thereby generating *N*-acylated peptide amphiphiles (lipopeptides; Fig. 4a). Specifically, the addition of subsequent Gly or other amino acids on the carboxyl terminus of NOG can produce *N*-acylated homo or heteropeptides, respectively (Fig. 4a) via acid-amine coupling (Fig. 4e). To test this, a mixture of 3 mM NOG and 200 mM Gly was subjected to a single wet-dry cycle of 24 h duration at pH 9.8 and 130 °C. These temperature, pH, and wet-dry cycling conditions are known to be suitable for peptide synthesis from non-activated Gly^3,25^. HRMS analysis showed the formation of lipopeptides with up to three Gly molecules added on to NOG (Fig. 4b and Supplementary Fig. 19). Next, we evaluated if the maximum length of the peptide chain generated on the NOG head group is influenced by temperature. It was observed that the maximum number of Gly additions on to the NOG increased with increasing temperature, with up to three additions observed at 130 °C, as compared to that at lower temperatures like 90 °C (Fig. 4c, Supplementary Figs. 19–24). A higher temperature of 140 °C did not facilitate further peptide extension (Supplementary Figure 24). Finally, we checked whether NOG could react with other amino acids like alanine, serine, and lysine to generate *N*-acylated heteropeptides. In these reactions, we observed only a single amino acid addition on to the NOG head group (Fig. 4d and Supplementary Figs. 25–27). Nonetheless, the lipopeptide formation efficiency seemed to vary based on the amino acid type (Supplementary Figs. 25–27). This may not be surprising given that the addition of an amino acid on the NOG head group involves a peptide bond formation, and Gly is known to react more efficiently as compared to other amino acids during abiotic peptide synthesis reactions^26,27^. In such reactions, the lower peptide yield of other amino acids like Ala as compared to that of Gly peptides has been attributed to the higher activation energy of peptide bond formation in Ala than in Gly^27^. It is plausible that the increase in the complexity and the bulkiness of side chains of amino acids other than Gly might reduce their overall efficiency to undergo abiotic oligomerization. Nonetheless, these results demonstrate NOG’s ability to react with different amino acids under wet-dry cycles, to generate a diverse set of lipopeptides. This diversity can be further increased in terms of the peptide sequence of these lipopeptides by using more than one amino acids in the starting reaction mixture. Increasing their sequence diversity might allow certain lipopeptides to acquire new advantageous properties like catalytic activity, which could provide them a selective advantage over other lipopeptides and for e.g. allow to become a part of emerging protocells.

## Conclusion

In this study, we establish NAAs as a promising model protoamphiphilic system. Specifically, we report a new prebiological route for their synthesis from an amino acid and a monoglyceride under wet-dry cycling conditions. This overcomes the dependence on structurally-complex diacyl lipids for their synthesis, and also strengthens their potential availability on early Earth. We also demonstrated the ability of NOG to generate vesicles from acidic to highly alkaline pH when admixed with GMO or OA. Notably, our recent study demonstrates that the addition of the GMO also stabilizes NOG vesicles in the presence of metal ions^28^. Together, these results highlight the potential of such heterogeneous amphiphilic systems in generating robust protocell compartments. These compartments could have supported a wide range of prebiotic reactions occurring at specific conditions of pH and metal ion concentrations. As far as the temperature is concerned, both the synthesis of NAAs and their vesicle formation and sustenance, requires a relatively high temperature (60–90 °C). On prebiotic Earth, such high temperature regimes would have been readily provided by terrestrial hot springs, which are also considered as a potential niche for life’s origin. Specific temperature regimes required for the synthesis and self-assembly process could have further been generated by the mixing of water from two source pools or at different sites within the same spring (origin versus run off site). Finally, we also showed that NOG could act as a substrate for peptide synthesis under wet-dry cycles, thereby generating different lipopeptides. Thus, we demonstrate the prebiotic availability of lipopeptides by illustrating their synthesis under prebiotically relevant conditions. Such membrane-anchored lipopeptides could have endowed early protocell membranes with new functions like catalytic activity^29^. Two major open questions in the origin of life research pertain to how early protocells would have acquired metabolism, and how would they have undergone reproduction in their entirety. The in-situ synthesis of catalytic lipopeptides may constitute a step towards understanding the role of membrane-residing catalysts in the emergence of early metabolism^30^. Notably, membrane adherence of short peptide catalysts, and the peptide products of such reactions have also been proposed to be useful for the onset of early protocellular reproduction^31^, and synchronizing growth of membrane and its molecular content^32^. Overall, our study indicates the pivotal role played by NAAs in shaping the emergence and evolution of protocell membranes on primitive Earth. It will be interesting to further explore how such lipoamino acids and lipopeptides could have contributed towards increasing protocellular stability, the emergence of stereoselectivity, and the onset of early metabolism via their catalytic activity.

## Methods

### Materials

Glycerol-1-monooleate and oleic acid were purchased from Nu-Chek-Prep (USA), while *N*-oleoyl glycine was purchased from Avanti polar lipids (USA). The rest of the chemicals and reagents, including amino acids and different buffers, were bought from Sigma-Aldrich (India). All the reagents and chemicals were bought in their most pure available form and used without further purification.

### Setting up *N*-acyl amino acid synthesis reaction under wet-dry cycles using GMO and an amino acid

Most of the protocol used in this study for NAA synthesis and analysis was adapted from our previously standardized protocol for NAA synthesis from phospholipids and amino acids^3^. In a typical reaction, adequate volumes of 10 mg/ml methanol stocks of GMO and OA were taken together in a glass vial, and methanol was evaporated under vacuum to form a dry lipid film. This film was then hydrated with 200 mM glycine (Gly) solution of pH 9.8, to get a final 200 µl reaction mixture containing 3 mM each of GMO and OA, and 200 mM Gly. OA was used in the reaction mixture to increase the overall solubility of GMO, which is otherwise poorly soluble in water. Given the buffering capacity of Gly between pH 8.6 to 10.6 (pKa of α-amino group of Gly is 9.6), a slightly higher concentration of Gly (200 mM) was used so that Gly can also maintain the reaction pH in addition to acting as one of the reactants. The reaction mixture was then subjected to three wet-dry cycles at 90 °C on a heating block (RCT basic, IKA). The duration of each wet-dry cycle was 24 hours, where water was allowed to evaporate to dryness at 90 °C, and this dried mixture was again rehydrated after 24 h with 200 µl of ultrapure water (18.2 MΩ-cm) to complete one wet-dry cycle. After rehydration, the solution was briefly vortexed to remix the reaction components. The same procedure was followed for subsequent wet-dry cycles. The final rehydration was done with 200 µl of ultrapure water two times with vortexing to recover the entire reaction content. This reaction solution was then stored at 4 °C until further analysis. Similar reaction conditions and experimental procedure was followed for NAA synthesis from GMO and other amino acids (alanine, valine, lysine, and serine), and also for the other control reactions, excepting for a few variations as mentioned. For the GMO + Lys reaction, pH was set to 9.1 to account for the lower pKa of the α-amino group of Lys (8.9). For the control reaction that did not contain Gly, a solution of GMO + OA (3 mM each) was prepared in 200 mM CHES buffer pH 9.8, which was subjected to three wet-dry cycles at 90 °C. For the OA + Gly control reaction, 3 mM OA solution was prepared in 200 mM Gly pH 9.8 and subjected to three wet-dry cycles at 90 °C.

### Monitoring the conversion of GMO to NOG using thin-layer chromatography

The conversion of GMO to NOG in the GMO + OA + Gly wet-dry cycling reaction was initially detected by TLC analysis on a normal phase silica plate (Merck), using a solvent system of toluene, chloroform, methanol (4:4:2) + 0.1 % glacial acetic acid. The reaction samples were loaded alongside commercially purchased standards of OA, GMO, and NOG for comparing the TLC spots. The solvent front was 6 cm. Also, the solvent system was run two times with intermediate drying for better resolution of TLC spots. Lipid species were visualized by staining with iodine. TLC analysis of other control reactions was also performed in a similar manner.

### Detection of NOG and other NAAs using high-resolution mass spectrometry

Firstly, the lipid content of the reaction mixture was separated from other aqueous content (unreacted Gly and any Gly oligomers generated during wet-dry cycling alongside NOG) using butanol extraction. Briefly, 200 µl of the wet-dry cycling reaction aliquot was further diluted by adding 100 µl of ultrapure water and to this 300 µl of 1-butanol (1:1 volume) was added. The solution was vortexed briefly and centrifuged at 13,000 rpm for 1 minute. The upper butanol layer was carefully removed without disturbing the aqueous layer, and collected separately. The remaining aqueous phase was again subjected to a second butanol extraction step as mentioned above. The butanol layers collected during both the extraction steps were mixed and the butanol was evaporated at 35 °C in a CentriVap DNA vacuum concentrator (Labconco) to form a dry lipid film. This lipid film was re-suspended into methanol and analyzed by HRMS (SYNAPT G2 High Definition Mass Spectrometer equipped with QTOF mass analyzer; Waters) by direct injection of the sample. Ionization was performed using the electrospray ionization (ESI) method in a negative ion mode with a capillary voltage of 3 kV. The NAAs generated by the reaction of GMO and other amino acids were also analyzed in a similar manner. For highly accurate mass detection (≤5 ppm), LockMass correction was applied using Leucine encephalin as an internal standard. The mass error was estimated using the following formula:

$${{{{{\rm{Mass}}}}}}\,{{{{{\rm{error}}}}}}\,({{{{{\rm{in}}}}}}\;{{{{{\rm{ppm}}}}}}\;{{{{{\rm{units}}}}}})= 	\, (({{{{{\rm{observed}}}}}}\;{{{{{\rm{mass}}}}}}-{{{{{\rm{calculated}}}}}}\; {{{{{\rm{mass}}}}}}) \\ 	 \div {{{{{\rm{calculated}}}}}}\;{{{{{\rm{mass}}}}}})* 1000000$$

### NMR analysis of NOG standard and reaction products

For the NMR analysis, reactions were set up in a bulk volume containing 6 ml of the starting reaction mixture, which was then split into 1 ml aliquots that were subjected to wet-dry cycling as mentioned above. NMR samples were prepared by extracting all the butanol layers from different aliquots of a single reaction in a single vial, followed by the evaporation of butanol to form a dry lipid film. This film was then dissolved in 600 µl of Methanol-d_4_ and subjected to ^1^H NMR analysis on a Bruker 400 MHz Spectrometer at IISER Pune NMR facility. The ^1^H NMR spectrum of the NOG standard was generated by directly dissolving 10 mg of commercially purchased NOG in 600 µl of Methanol-d_4_, and subjected to ^1^H NMR analysis as mentioned above. The NMR results were analyzed using Mnova software from Mestrelab.

### Vesicle formation by NOG-based amphiphile systems

For NOG + GMO mixed system, appropriate volumes of NOG and GMO methanol stocks (10 mg/ml each) were taken together in a microfuge tube and mixed with 10 µl of 50 µM R18 dye (see below). The methanol was evaporated under vacuum at 35 °C to form a dry lipid film, which was hydrated with 50 µl of 200 mM buffer of a particular pH to get a 6 mM total amphiphile concentration, with NOG and GMO in a 2:1 ratio. Solutions of different pH values were prepared in ultrapure water (18.2 MΩ-cm) using the following buffers: acetate for pH 4 and 5, MES for pH 6, HEPES for pH 7, bicine for pH 8 and 9, CHES for pH 10, and CAPS for pH 11. After hydration of the lipid film with a given buffer, the resultant solution was then incubated at 60 °C on ThermoMixer C (Eppendorf) for one hour duration with constant shaking at 500 rpm, along with frequent mixing by vortexing and pipetting. This heating step is important for the generation of vesicles by NOG-based amphiphile systems, because the chain-melting transition temperature of NOG is higher than room temperature^3^. Also, this high temperature should be maintained to keep NOG in a vesicle form^3,28^. This was achieved by incubating vesicle solutions in ThermoMixer C with a temperature controlling lid (ThermoTop; Eppendorf) that allowed to maintain a constant temperature, both inside and outside of the microfuge tube, thereby avoiding volume changes due to evaporation and condensation. A similar procedure was followed for generating vesicles from NOG, OA, and NOG + OA mixed systems. The total amphiphile concentration was 6 mM for all these systems, with NOG and OA being in a 1:1 ratio (that is 3 mM each) in the mixed system.

### Microscopic analysis of vesicles generated by NOG-based amphiphile systems

Vesicles were visualized using differential interference contrast (DIC) and fluorescence microscopy (Axio Imager Z1, Carl Zeiss) under 40X objective (NA = 0.75). For fluorescence-based imaging, vesicles were stained with 10 µM Octadecyl Rhodamine B Chloride (R18) (Invitrogen), which is an amphiphilic dye that specifically gets inserted into the membrane bilayer and allows a better visualization of vesicles having different size, shape, and lamellarity. For staining vesicles with R18 dye, 10 µl of 50 µM methanol stock of R18 was added during the mixing of methanol stocks of amphiphiles, to get a final 10 µM concentration of R18 in the 50 µl vesicle solution. These vesicles were then visualized by fluorescence microscopy using the filter set 43 HE (Ex: 550/25 nm, Em: 605/70 nm, Beamsplitter: FT 570). Image acquisition was done using AxioVision software and the acquired images were further processed using ImageJ.

### Setting up the reaction for peptide chain growth on the NOG surface

In a typical reaction, appropriate volume of 10 mg/ml methanol stock of NOG was taken in a glass vial, and methanol was evaporated under vacuum to prepare a dry lipid film of NOG. This film was then hydrated with 200 µl of 200 mM of amino acid solution of pH 9.8 (except for lysine where the pH was 9.1), to get a 3 mM NOG solution, which was then subjected to a single wet-dry cycle of 24 hours duration at 130 °C. These conditions were adapted from previously standardized conditions^3,25^ for peptide synthesis from non-activated amino acids, as the addition of subsequent amino acids on the NOG terminus involves the formation of a peptide bond. In temperature variation experiments for NOG and Gly, the wet-dry cycling temperature was varied from 90 °C to 140 °C. The rehydration was done twice with 200 µl ultrapure water to recover the entire reaction content, which was then subjected to butanol extraction as mentioned above to extract the total lipid content of the reaction (unreacted NOG and *N*-oleoyl peptide products). This lipid content was analyzed using HRMS via direct injection of the sample as detailed above, but in a positive mode, which was found to be more suitable for the detection of *N*-oleoyl peptide products as compared to the negative mode. Both NOG and its extension products were mostly detected as sodium adducts.

All the experiments in this study were performed in at least two independent replicates.

### Reporting summary

Further information on research design is available in the Nature Research Reporting Summary linked to this article.

## Supplementary information

Rajamani_PR File

Supplementary Information

Reporting Summary

## Data Availability

All data generated or analyzed during this study are included in this published article (and its supplementary information files).
