# Supplementary material for: Elucidating N-acyl amino acids as a model protoamphiphilic system
Source: Commun Chem. 2022 Nov 9;5:147. doi: 10.1038/s42004-022-00762-9 (PMC9814278; doi:10.1038/s42004-022-00762-9)
Supplement: Supplementary file 2 — Supplementary Information [file 42004_2022_762_MOESM2_ESM.pdf]

Supplementary Information

Elucidating N-acyl amino acids as a model protoamphiphilic system

Manesh Prakash Joshi[1*], Ashwin Uday[1], Sudha Rajamani[1*]

[1]Department of Biology, Indian Institute of Science Education and Research Pune, Pune 411008, India.

[*]Corresponding authors: M.P.J. (manesh.joshi@students.iiserpune.ac.in),
S.R. (srajamani@iiserpune.ac.in)
Phone (O): +91 – 020 – 25908061
Fax (O): +91 – 020 – 25899790

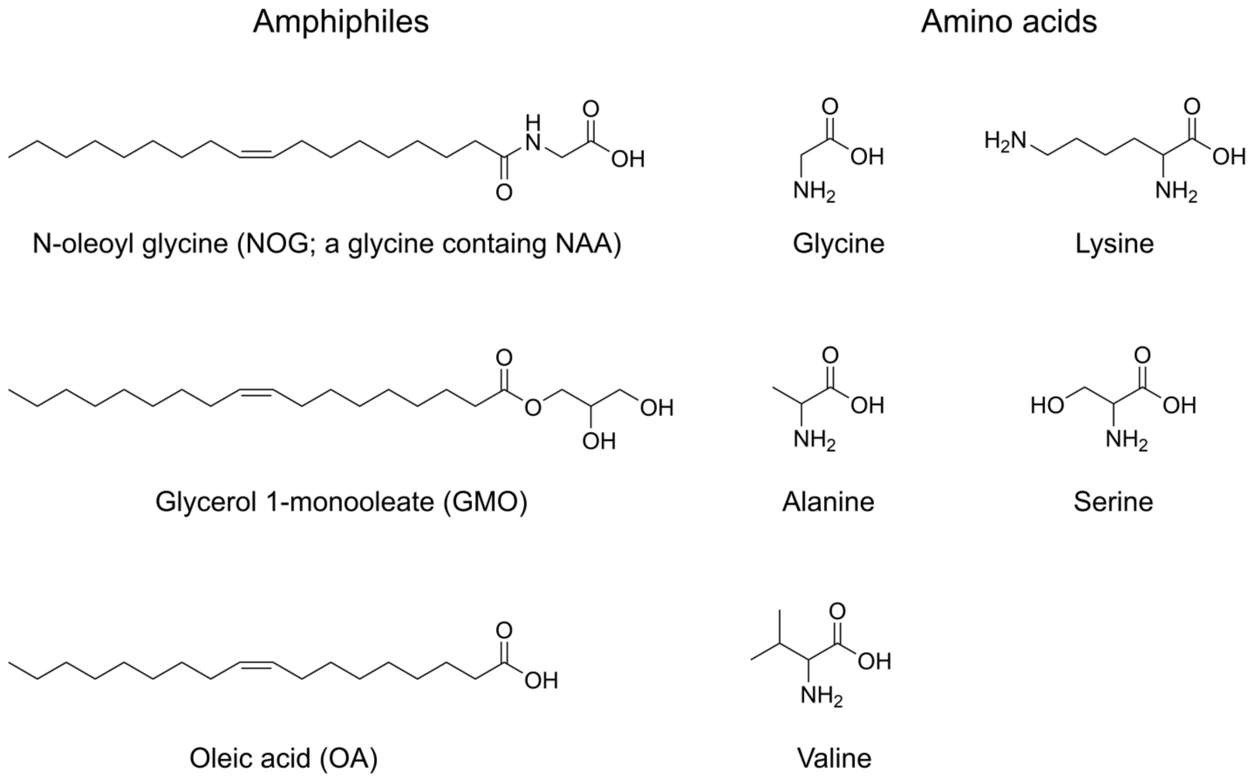

Supplementary Figure 1. Different amphiphiles and amino acids that were used in this study.

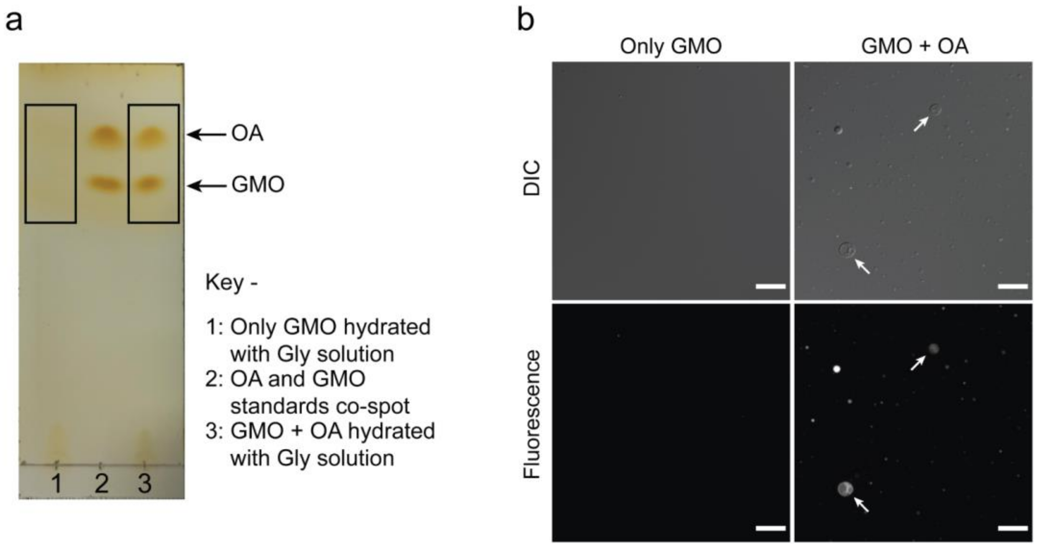

Supplementary Figure 2. OA increases the solubility of GMO in water via vesicle formation. Dry lipid films of only GMO and GMO + OA were hydrated with 200 mM Gly pH 9.8 to prepare solutions containing either GMO (3 mM) or a mixture of GMO and OA (3 mM each). **a)** The TLC analysis of the resultant solutions shows that GMO gets solubilized in water in the presence of OA (Lane 3), but not on its own (Lane 1) (as highlighted by black square boxes). **b)** The microscopic analysis of the same solutions in a), reveals that a solution containing GMO + OA has the presence of vesicles (indicated by white arrows). However, vesicles are absent in the case of only GMO solution. For fluorescence imaging, vesicles were stained with 10 µM R18 dye. The scale bar is 20 µm.

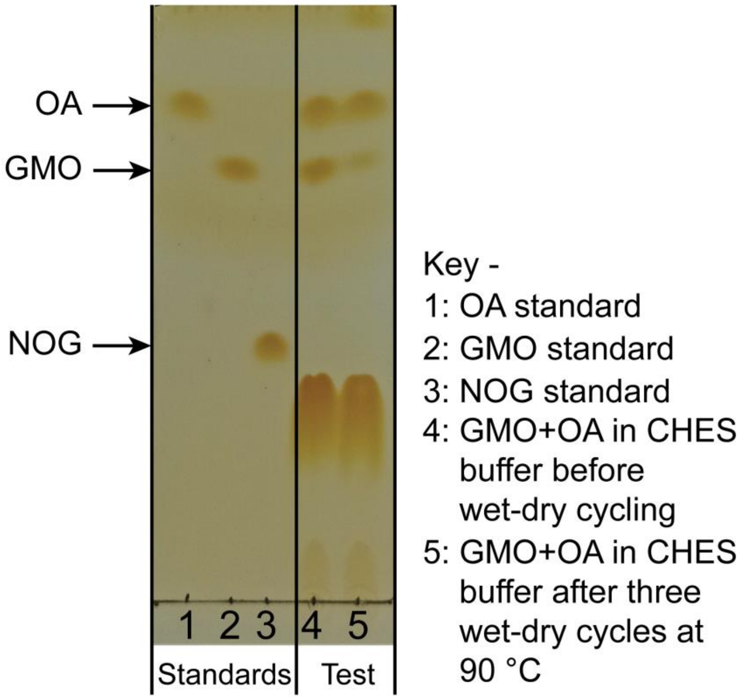

Supplementary Figure 3. GMO + OA mixture in the absence of Gly does not form NOG. The reaction was set up in 200 mM CHES buffer pH 9.8 and subjected to three wet-dry cycles at 90 °C. The spot corresponding to NOG did not appear after wet-dry cycling (compare lanes 3 and 5). The intensity of the GMO spot also decreased after wet-dry cycling, potentially indicating the degradation of GMO due to alkaline hydrolysis. The spot present in both lanes 4 and 5 at the sample front (towards the bottom of the TLC) is likely that of CHES buffer.

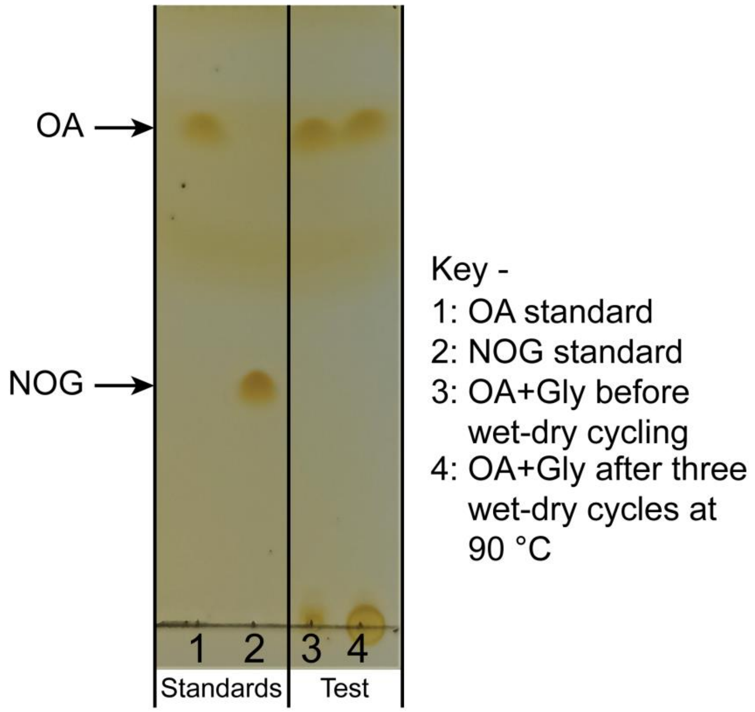

Supplementary Figure 4. Only OA in the presence of Gly does not form NOG. A TLC analysis of the reaction containing 3 mM OA in 200 mM Gly pH 9.8, which was subjected to three wet-dry cycles at 90 °C, did not show the NOG spot after wet-dry cycling (compare lanes 2 and 4), indicating that OA itself does not react with Gly to form NOG under these reaction conditions.

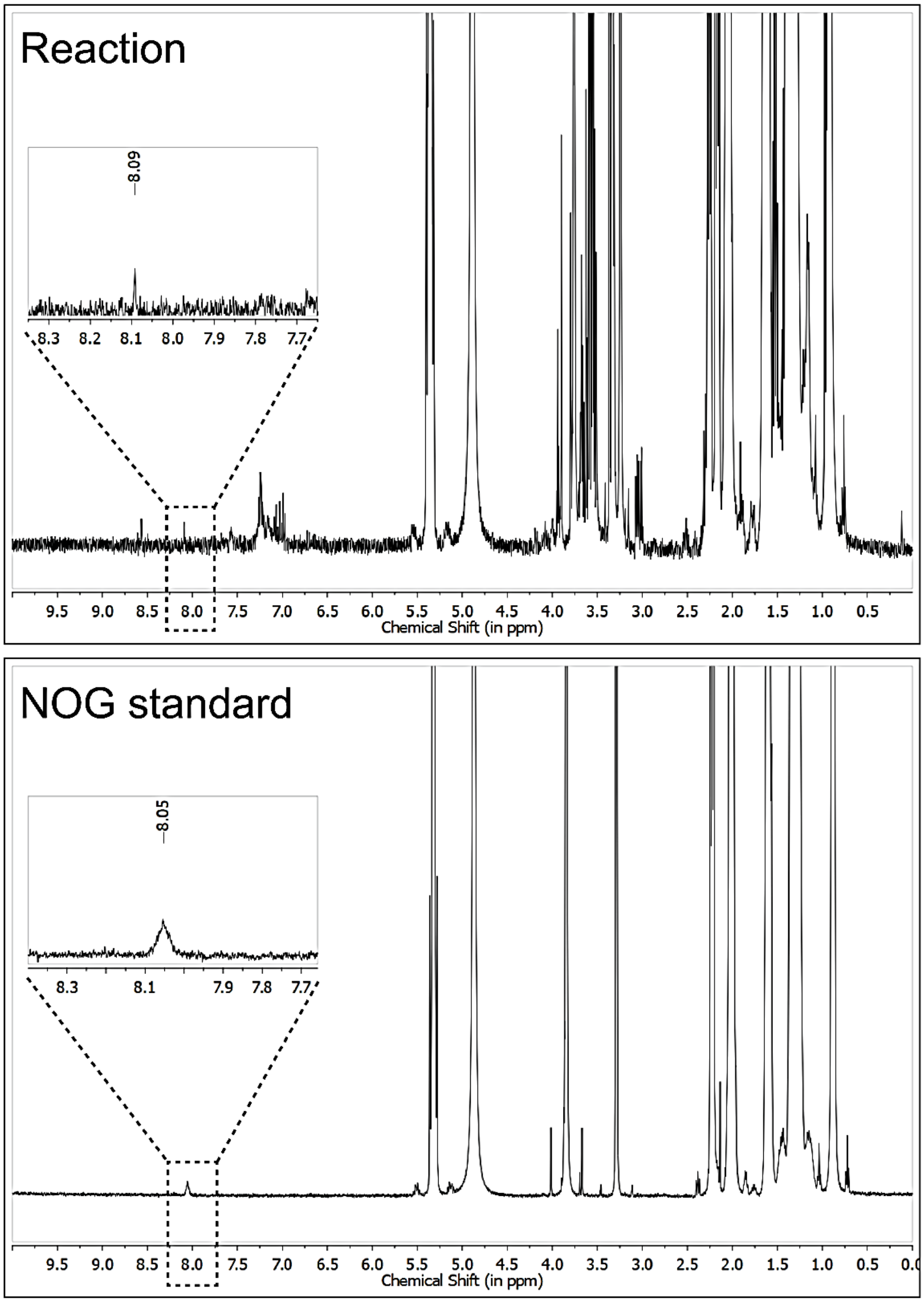

Supplementary Figure 5. ^{1}H NMR analysis of the GMO + OA + Gly wet-dry cycling reaction. The final lipid content of GMO + OA + Gly wet-dry cycling reaction (top panel), shows a signature peak for the amide hydrogen of NOG at 8.09 ppm (see inset), which is comparable to the one observed for the NOG standard (bottom panel) at 8.05 ppm (see inset). The solvent used was methanol-d_4.

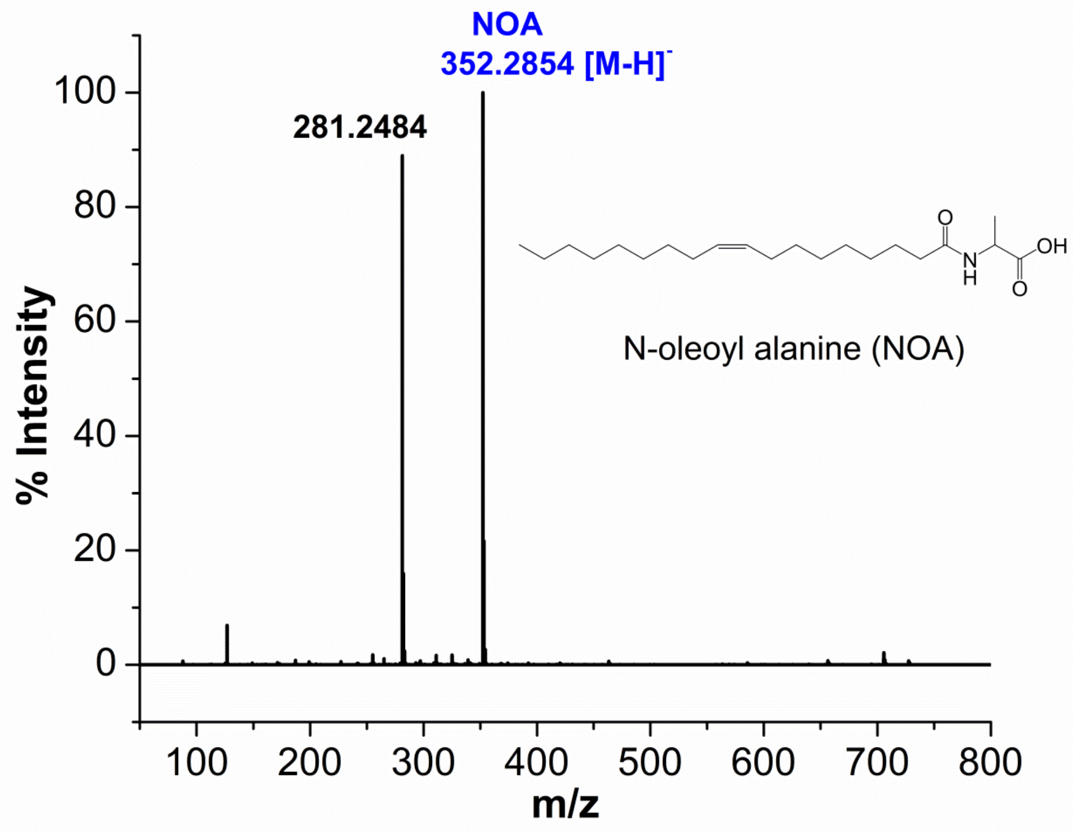

Supplementary Figure 6. Formation of N-oleoyl alanine (NOA) in the GMO + OA + alanine wet-dry cycling reaction. HRMS analysis (negative mode) of the lipid content of the final reaction mixture showed a peak for NOA (calculated: 352.2857; observed: 352.2854; mass error = − 0.9 ppm). Another peak at 281.24 corresponds to OA.

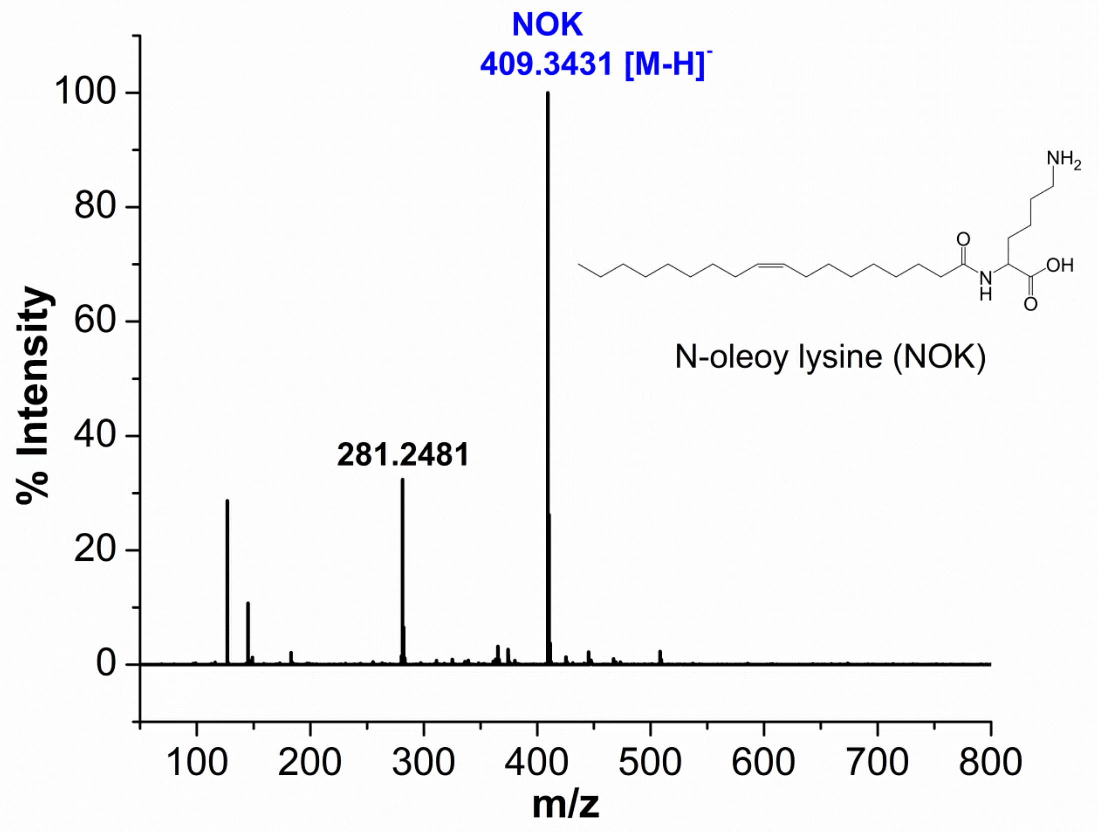

Supplementary Figure 7. Formation of N-oleoyl lysine (NOK) in the GMO + OA + lysine wet-dry cycling reaction. HRMS analysis (negative mode) of the lipid content of the final reaction mixture showed a peak for NOK (calculated: 409.3436; observed: 409.3431; mass error = − 1.2 ppm). Another peak at 281.24 corresponds to OA.

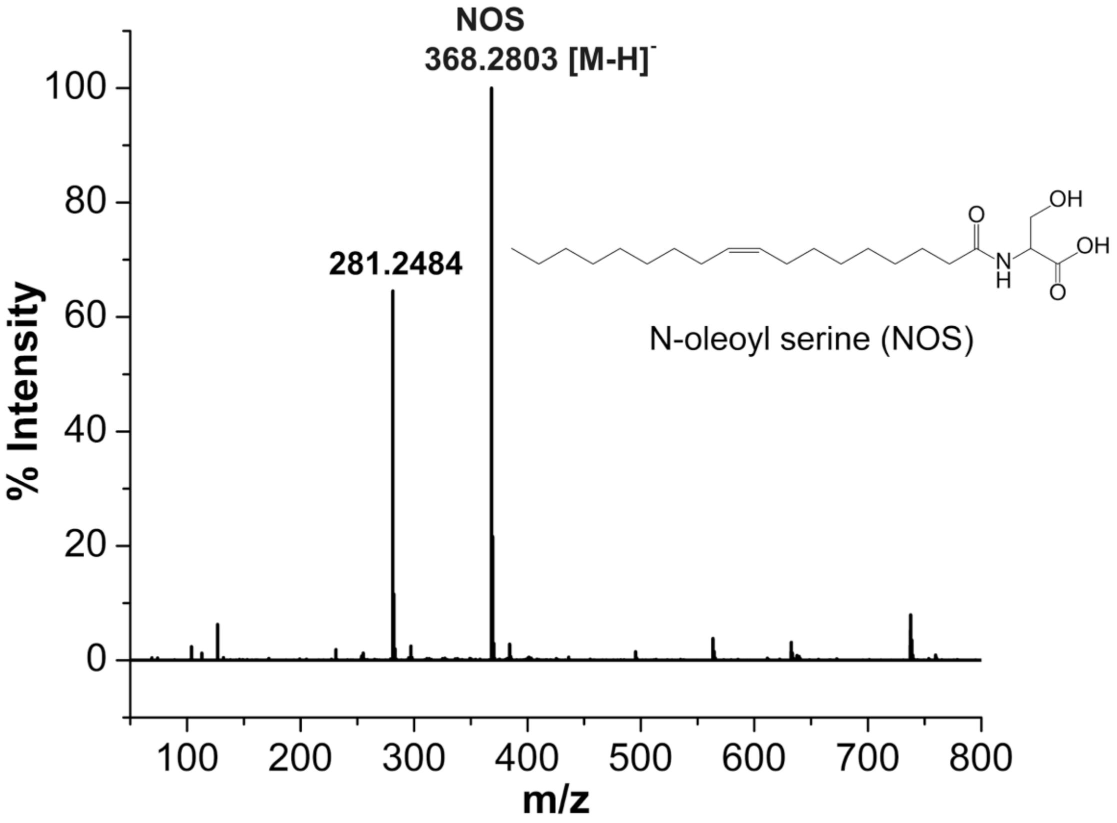

Supplementary Figure 8. Formation of N-oleoyl serine (NOS) in the GMO + OA + serine wet-dry cycling reaction. HRMS analysis (negative mode) of the lipid content of the final reaction mixture showed a peak for NOS (calculated: 368.2806; observed: 368.2803; mass error = − 0.8 ppm). Another peak at 281.24 corresponds to OA.

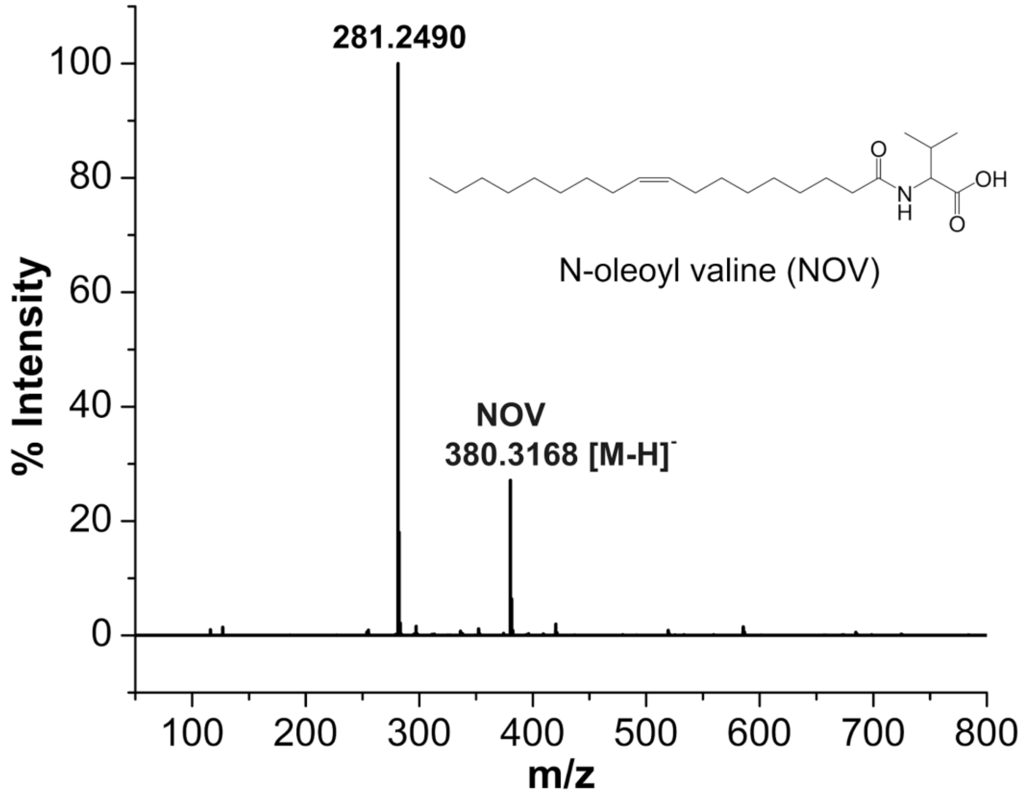

Supplementary Figure 9. Formation of N-oleoyl valine (NOV) in the GMO + OA + valine wet-dry cycling reaction. HRMS analysis (negative mode) of the lipid content of the final reaction mixture showed a peak for NOV (calculated: 380.317; observed: 380.3168; mass error = − 0.5 ppm). Another peak at 281.24 corresponds to OA.

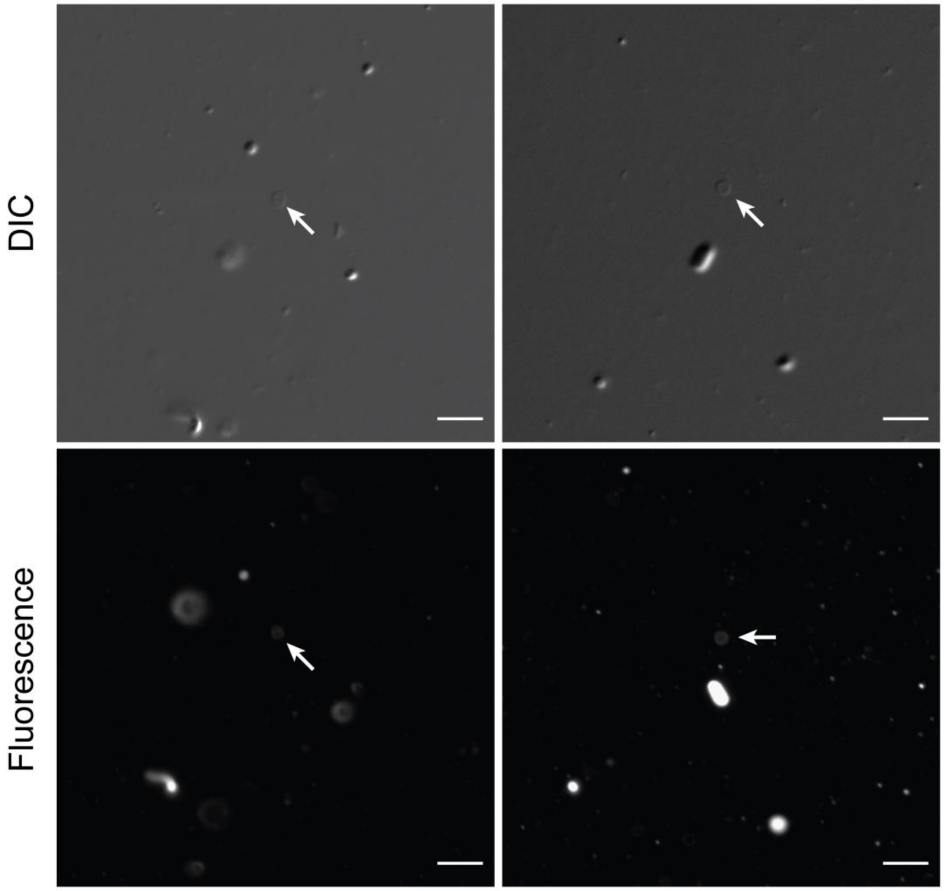

Supplementary Figure 10. Additional microscopy images for vesicle formation by NOG + GMO (6 mM; 2:1 ratio) mixed system at pH 4. Representative vesicle structures are indicated by white arrows. For fluorescence imaging, vesicles were stained with 10 μM R18 dye. The scale bar is 10 μm.

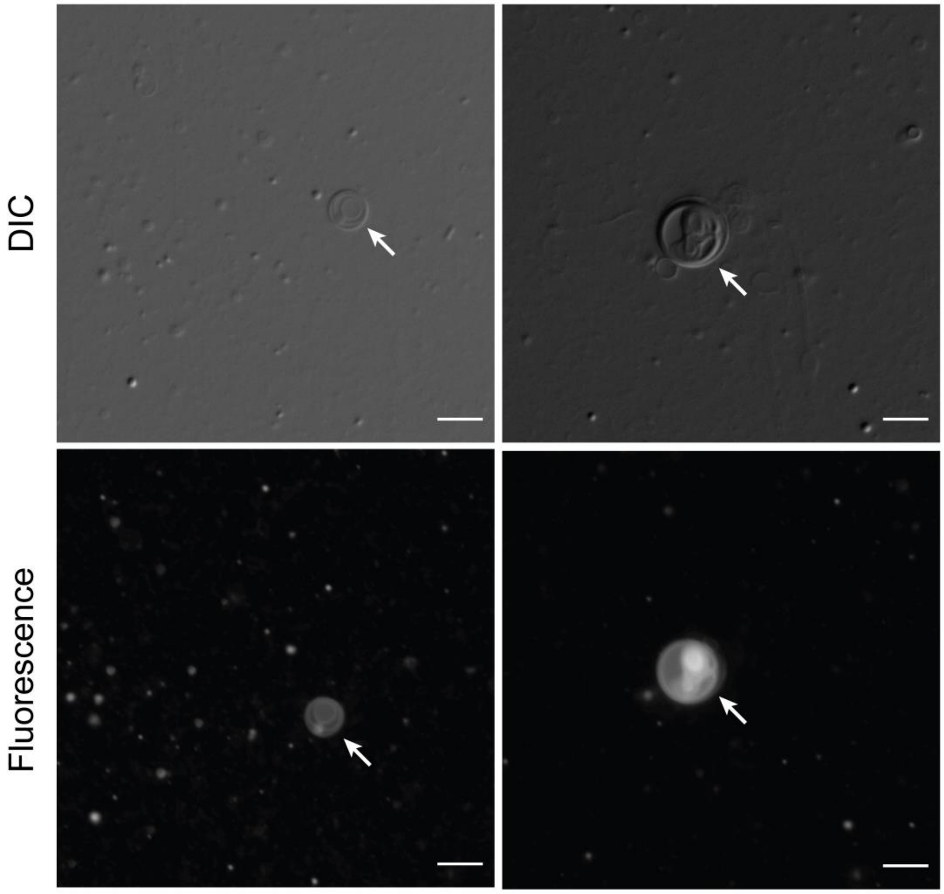

Supplementary Figure 11. Additional microscopy images for vesicle formation by NOG + GMO (6 mM; 2:1 ratio) mixed system at pH 5. Representative vesicle structures are indicated by white arrows. For fluorescence imaging, vesicles were stained with 10 μM R18 dye. The scale bar is 10 μm.

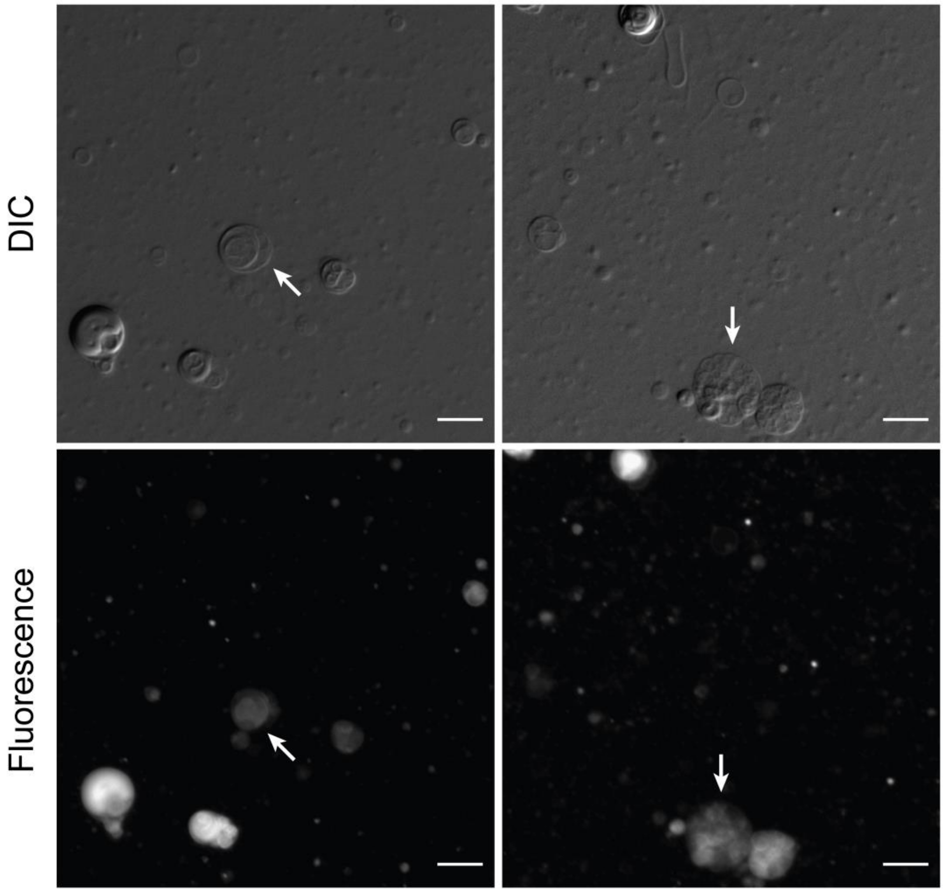

Supplementary Figure 12. Additional microscopy images for vesicle formation by NOG + GMO (6 mM; 2:1 ratio) mixed system at pH 6. Representative vesicle structures are indicated by white arrows. For fluorescence imaging, vesicles were stained with 10 μM R18 dye. The scale bar is 10 μm.

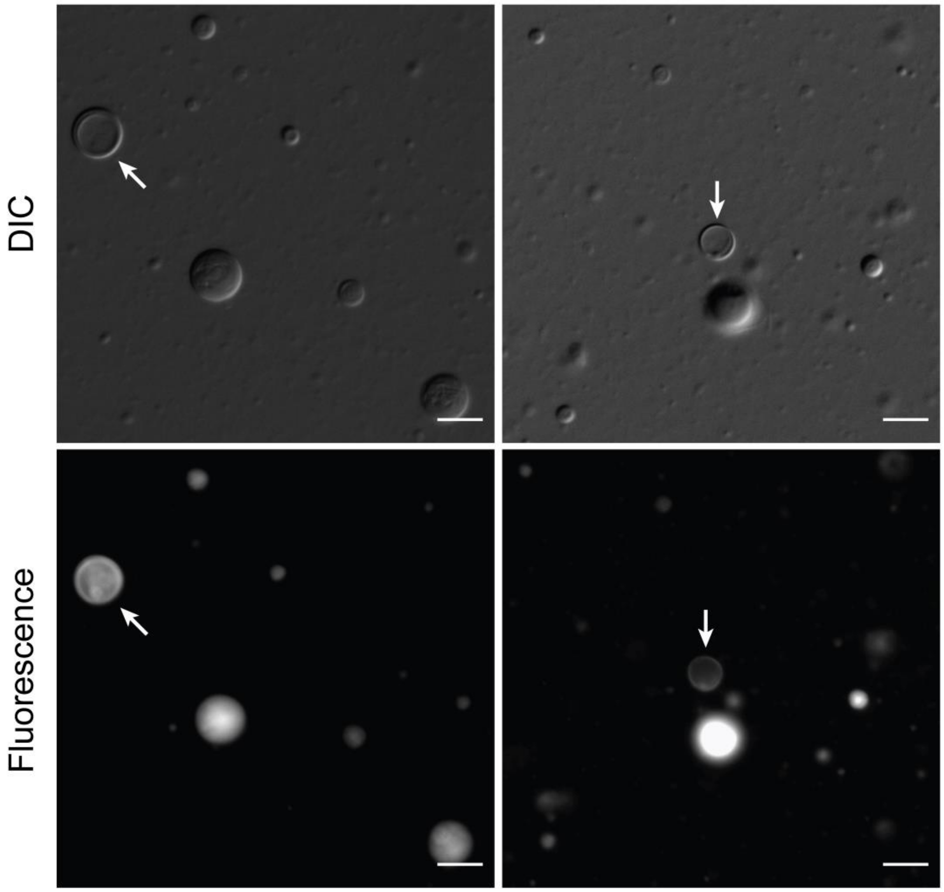

Supplementary Figure 13. Additional microscopy images for vesicle formation by NOG + GMO (6 mM; 2:1 ratio) mixed system at pH 7. Representative vesicle structures are indicated by white arrows. For fluorescence imaging, vesicles were stained with 10 μM R18 dye. The scale bar is 10 μm.

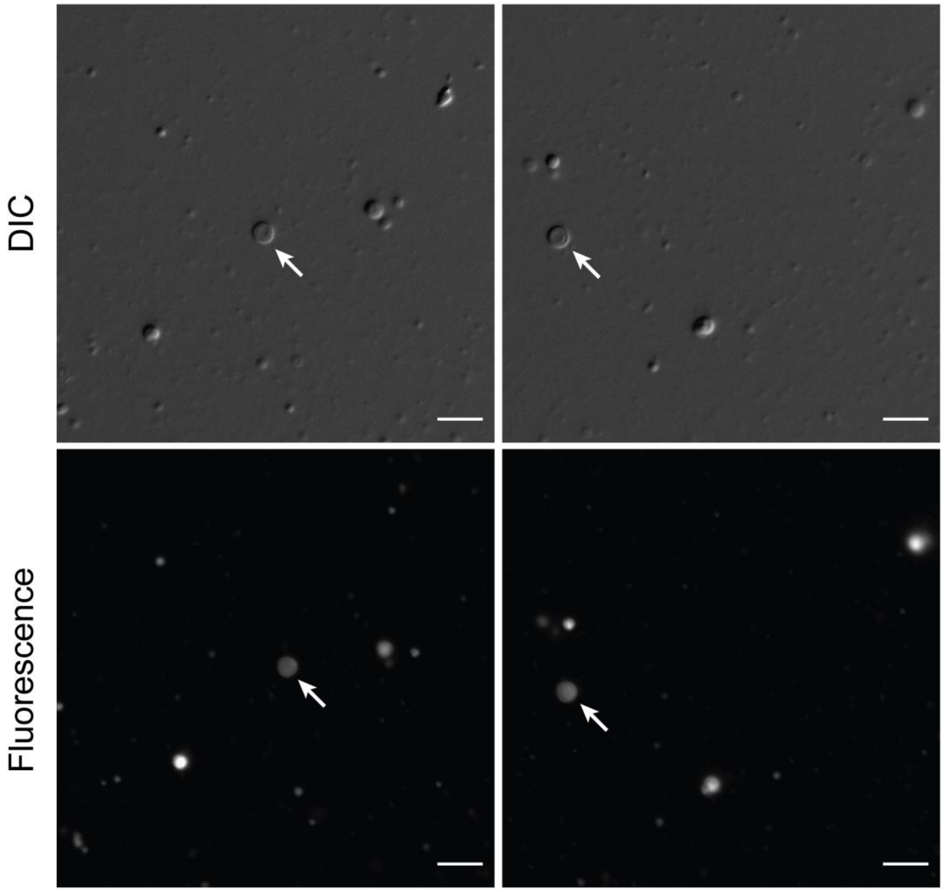

Supplementary Figure 14. Additional microscopy images for vesicle formation by NOG + GMO (6 mM; 2:1 ratio) mixed system at pH 8. Representative vesicle structures are indicated by white arrows. For fluorescence imaging, vesicles were stained with 10 μM R18 dye. The scale bar is 10 μm.

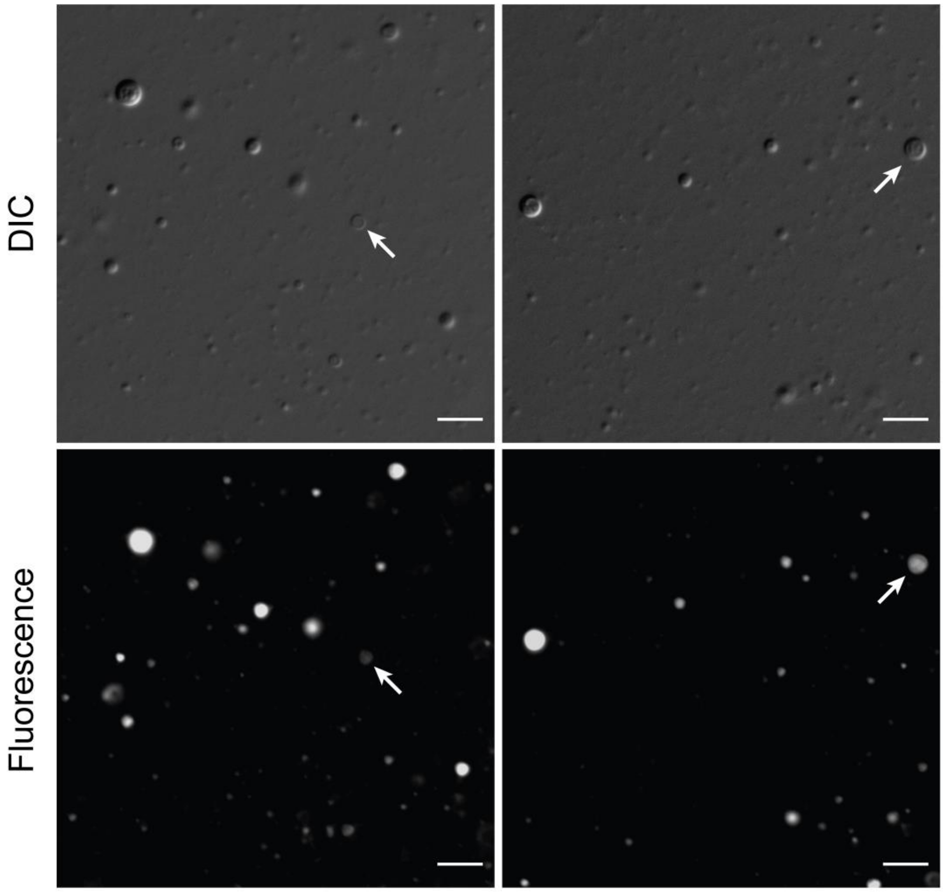

Supplementary Figure 15. Additional microscopy images for vesicle formation by NOG + GMO (6 mM; 2:1 ratio) mixed system at pH 9. Representative vesicle structures are indicated by white arrows. For fluorescence imaging, vesicles were stained with 10 µM R18 dye. The scale bar is 10 µm.

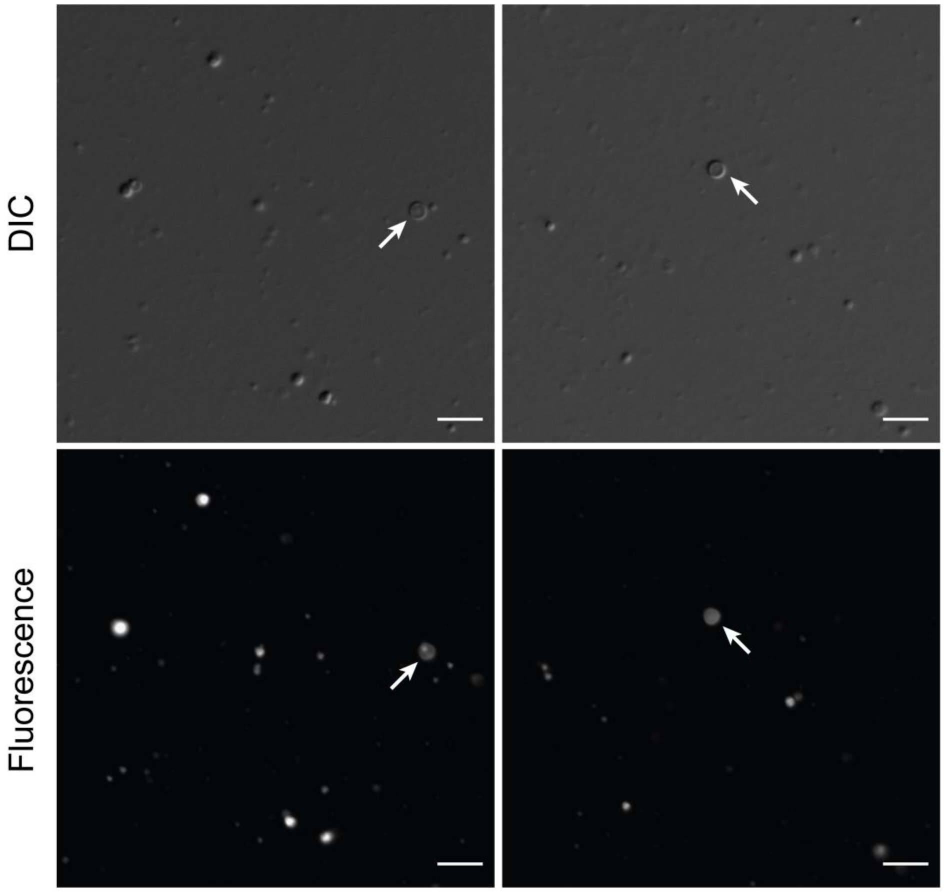

Supplementary Figure 16. Additional microscopy images for vesicle formation by NOG + GMO (6 mM; 2:1 ratio) mixed system at pH 10. Representative vesicle structures are indicated by white arrows. For fluorescence imaging, vesicles were stained with 10 µM R18 dye. The scale bar is 10 µm.

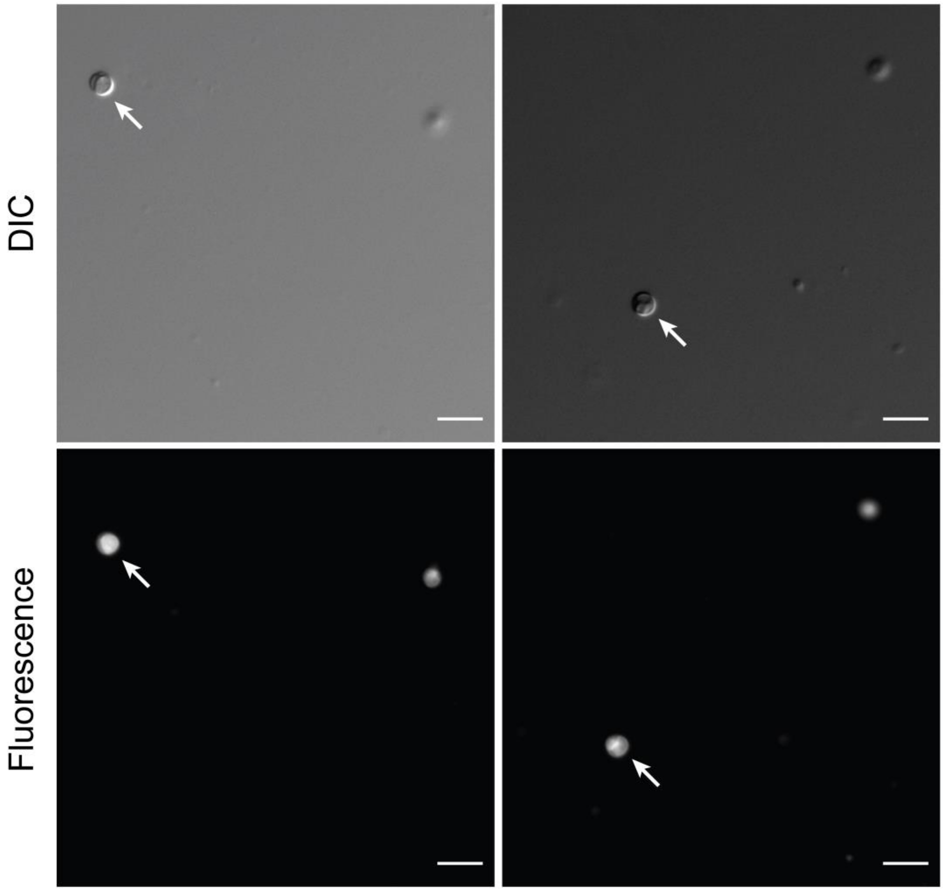

Supplementary Figure 17. Additional microscopy images for vesicle formation by NOG + GMO (6 mM; 2:1 ratio) mixed system at pH 11. Representative vesicle structures are indicated by white arrows. For fluorescence imaging, vesicles were stained with 10 μM R18 dye. The scale bar is 10 μm.

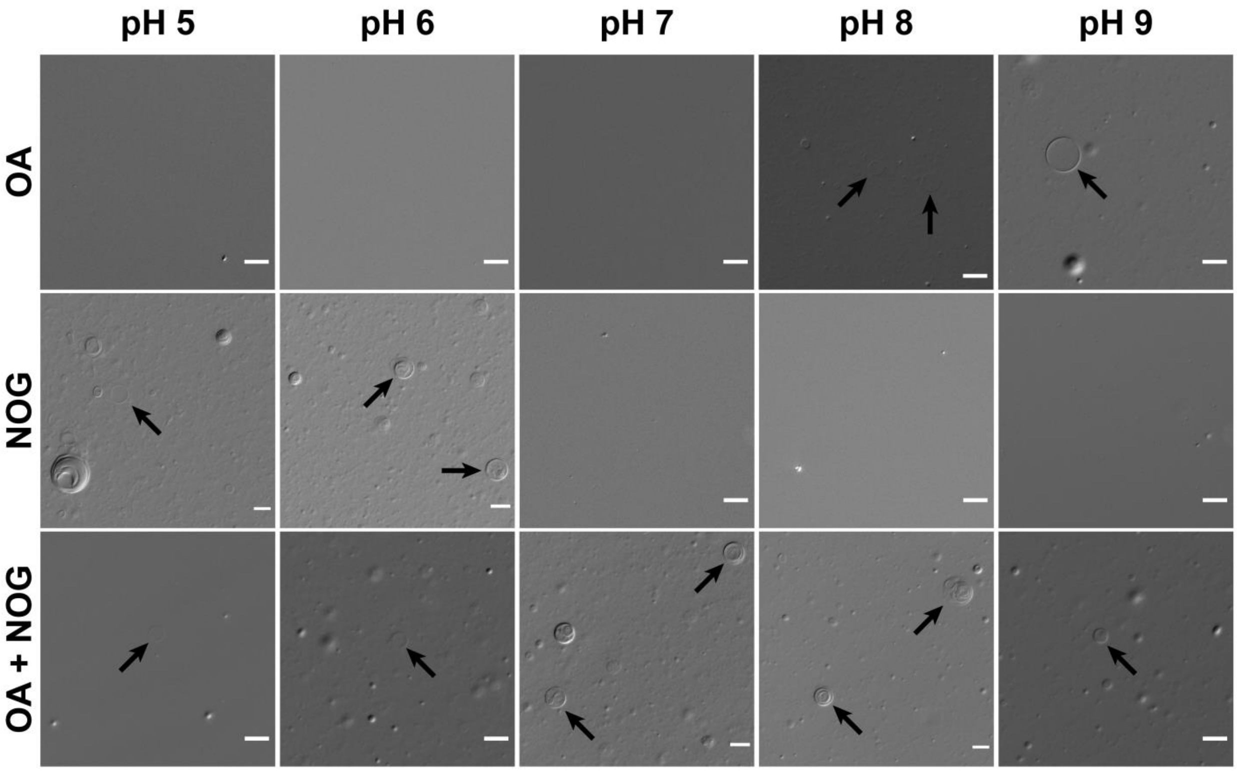

Supplementary Figure 18. DIC microscopy images for vesicle formation behavior of NOG + OA (6 mM; 1:1 ratio) mixed system that is shown in Figure 3. Representative vesicle structures are indicated by black arrows. The scale bar is 10 µm.

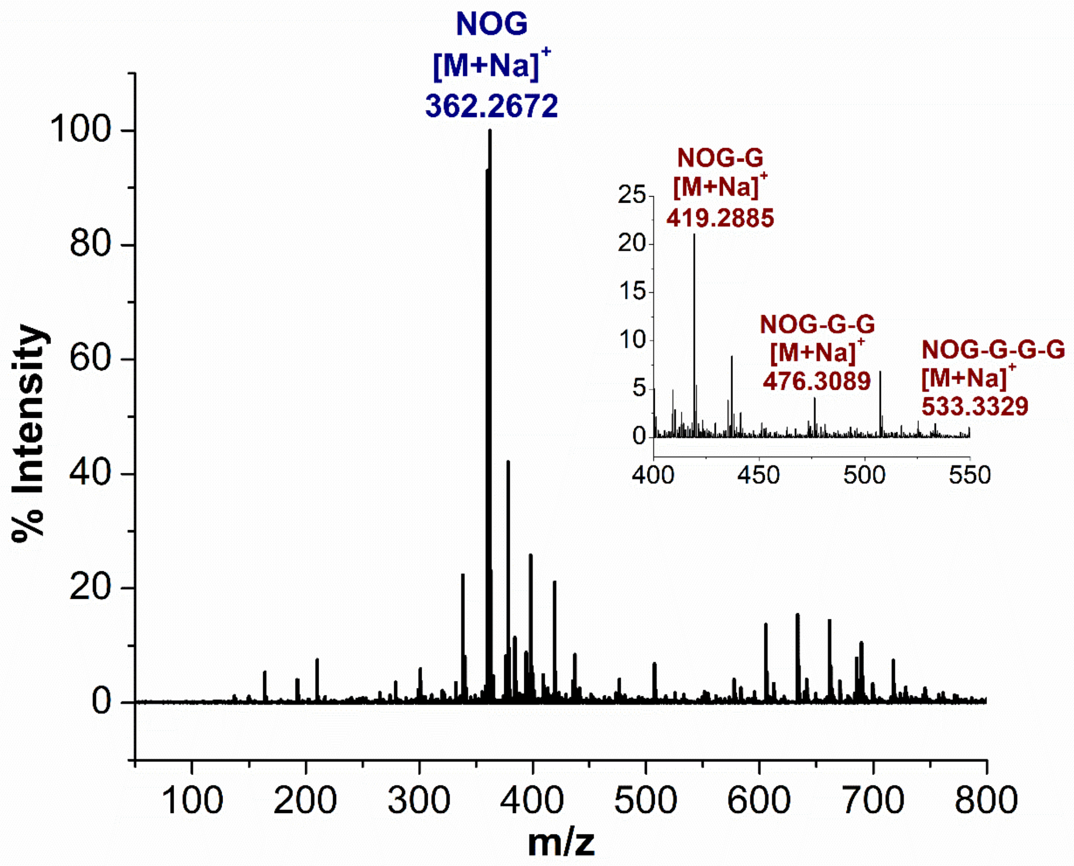

Supplementary Figure 19. HRMS analysis (positive mode) of the final lipid content of NOG + Gly wet-dry cycling reaction at 130 °C. The HRMS spectrum shows peaks for sodiated adducts of NOG and the peptide chain extension products (see inset). Up to three Gly additions were observed on the NOG surface, which are indicated by NOG-G for a single addition (cal: 419.288; obs: 419.2885; mass error = 1.2 ppm), NOG-G-G for two additions (cal: 476.3095; obs: 476.3089; mass error = − 1.3 ppm), and NOG-G-G-G for three additions (cal: 533.331; obs: 533.3329; mass error = 3.6 ppm).

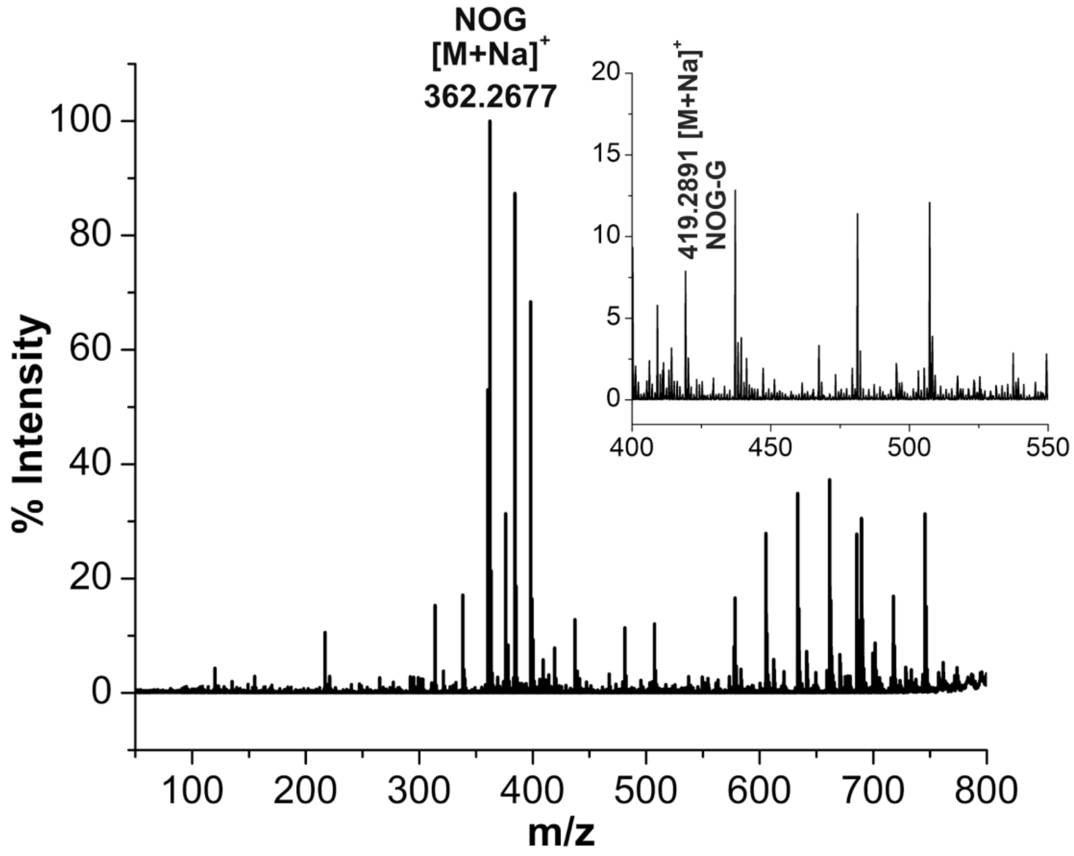

Supplementary Figure 20. HRMS analysis (positive mode) of the final lipid content of NOG + Gly wet-dry cycling reaction at 90 °C. The HRMS spectrum shows peaks for sodiated adducts of NOG and the peptide chain extension product (see inset). Only a single Gly addition was observed on the NOG surface at 90 °C, which is indicated by NOG-G (cal: 419.288; obs: 419.2891; mass error = 2.6 ppm).

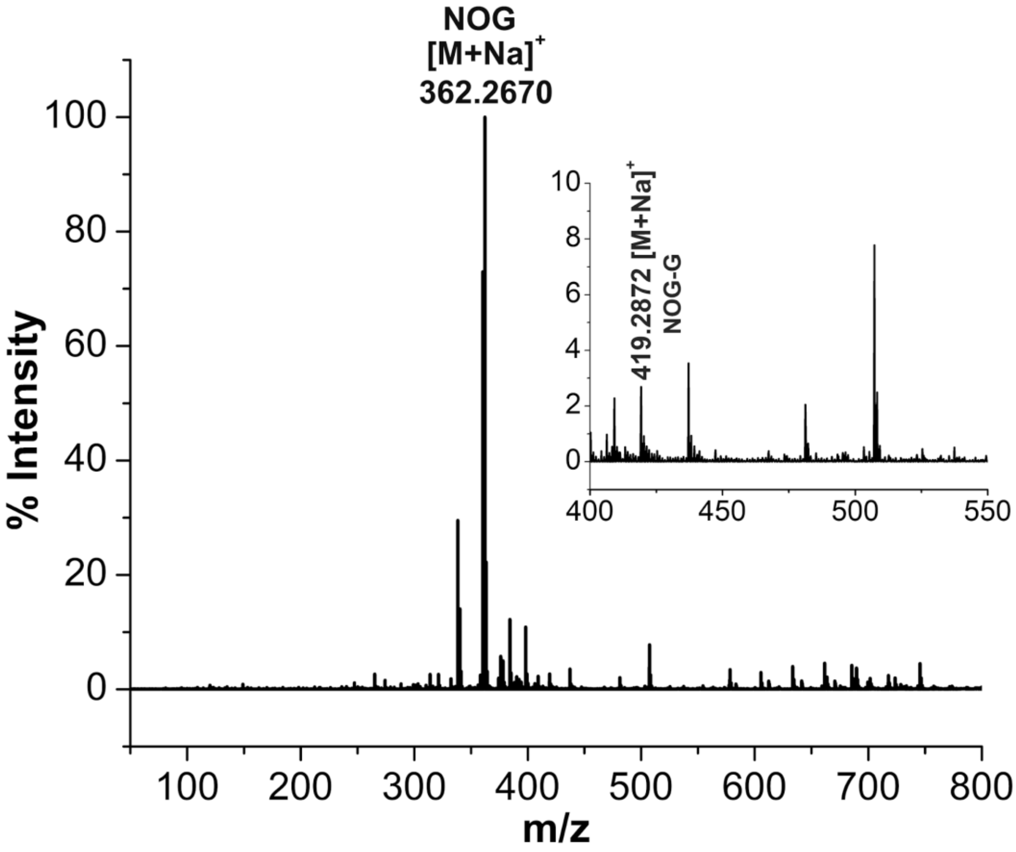

Supplementary Figure 21. HRMS analysis (positive mode) of the final lipid content of NOG + Gly wet-dry cycling reaction at 100 °C. The HRMS spectrum shows peaks for sodiated adducts of NOG and the peptide chain extension product (see inset). Only a single Gly addition was observed on the NOG surface at 100 °C, which is indicated by NOG-G (cal: 419.288; obs: 419.2872; mass error = − 1.9 ppm).

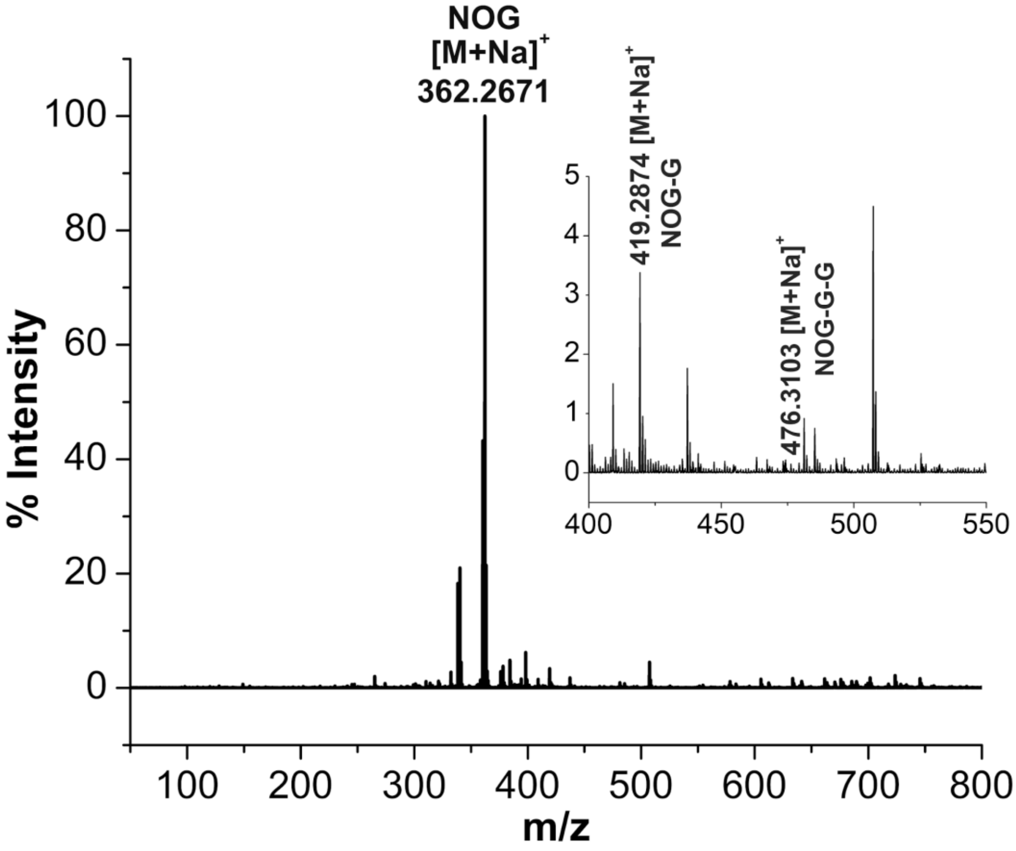

Supplementary Figure 22. HRMS analysis (positive mode) of the final lipid content of NOG + Gly wet-dry cycling reaction at 110 °C. The HRMS spectrum shows peaks for sodiated adducts of NOG and the peptide chain extension products (see inset). Up to two Gly additions were observed on the NOG surface at 110 °C, which are indicated by NOG-G for a single addition (cal: 419.288; obs: 419.2874; mass error = − 1.4 ppm), and NOG-G-G for two additions (cal: 476.3095; obs: 476.3103; mass error = 1.7 ppm).

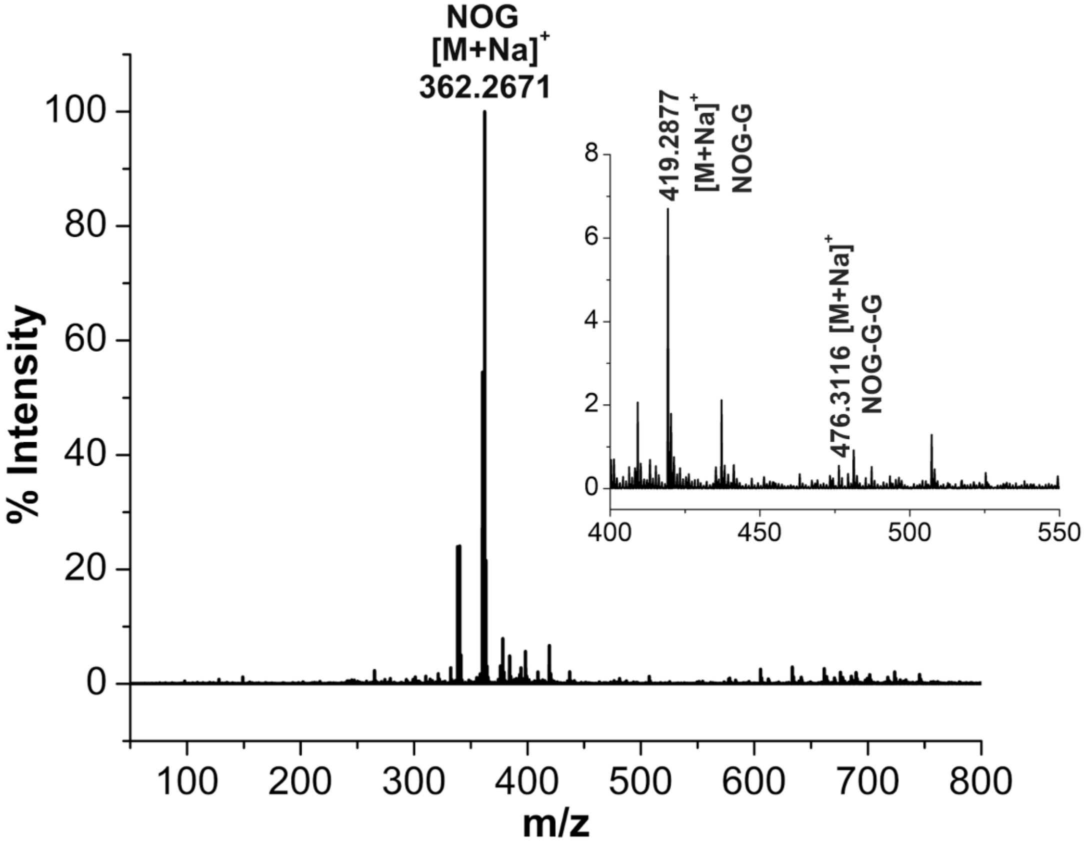

Supplementary Figure 23. HRMS analysis (positive mode) of the final lipid content of NOG + Gly wet-dry cycling reaction at 120 °C. The HRMS spectrum shows peaks for sodiated adducts of NOG and the peptide chain extension products (see inset). Up to two Gly additions were observed on the NOG surface at 120 °C, which are indicated by NOG-G for a single addition (cal: 419.288; obs: 419.2877; mass error = − 0.7 ppm), and NOG-G-G for two additions (cal: 476.3095; obs: 476.3116; mass error = 4.4 ppm).

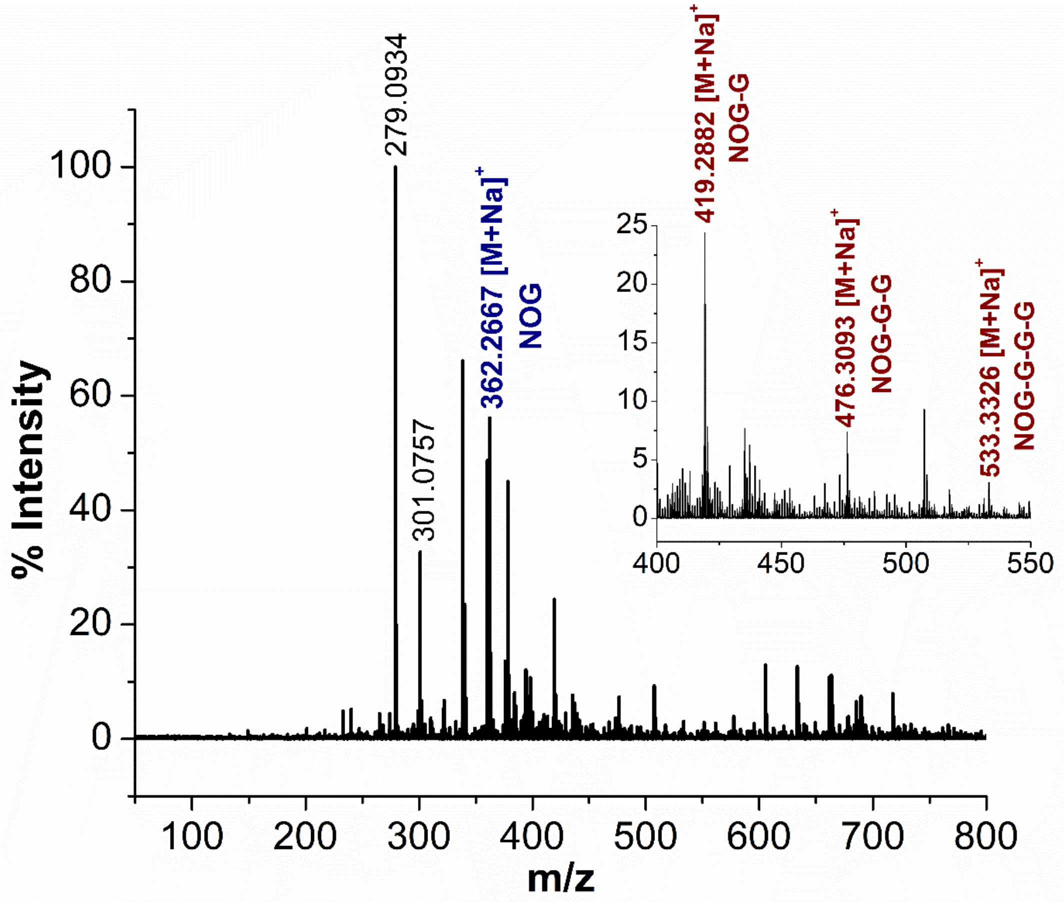

Supplementary Figure 24. HRMS analysis (positive mode) of the final lipid content of NOG + Gly wet-dry cycling reaction at 140 °C. The HRMS spectrum shows peaks for sodiated adducts of NOG and the peptide chain extension products (see inset). Up to three Gly additions were observed on the NOG surface at 140 °C, which are indicated by NOG-G for a single addition (cal: 419.288; obs: 419.2882; mass error = 0.5 ppm), NOG-G-G for two additions (cal: 476.3095; obs: 476.3093; mass error = − 0.4 ppm), and NOG-G-G-G for three additions (cal: 533.331; obs: 533.3326; mass error = 3.0 ppm). Also, there were some predominant stray peaks like 279.09 and 301.07 in the lower mass region of the spectrum, potentially indicating the breakdown of NOG at 140 °C.

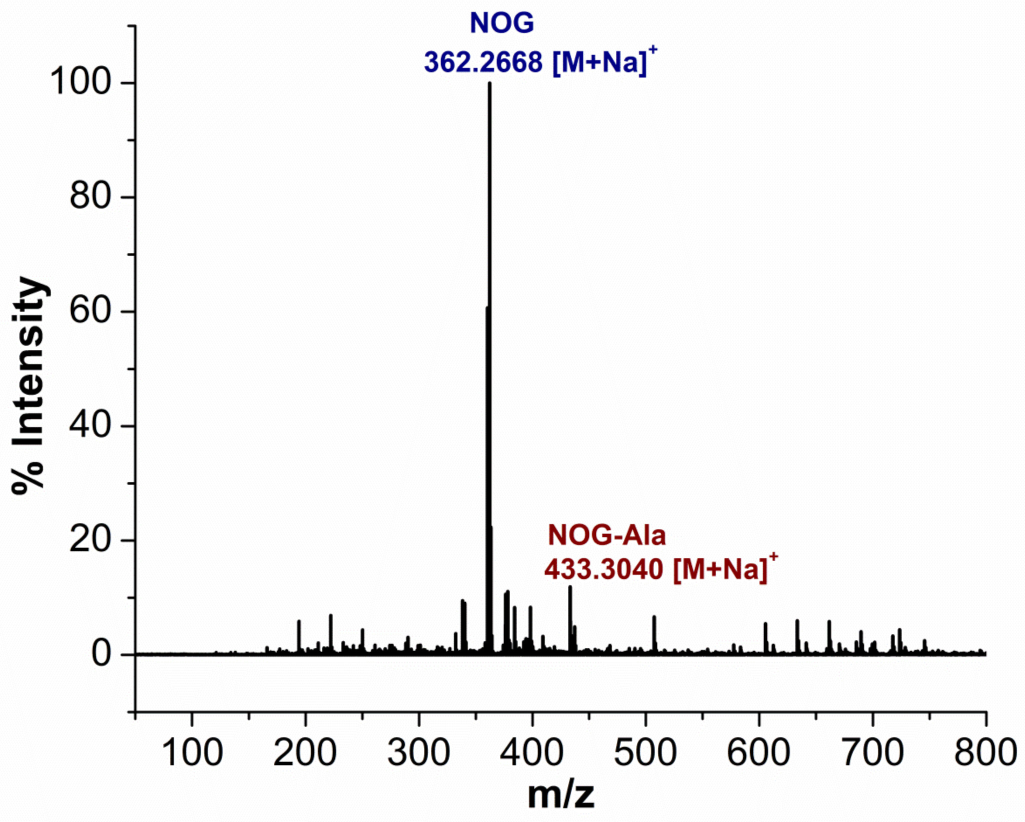

Supplementary Figure 25. HRMS analysis (positive mode) of the final lipid content of NOG + alanine wet-dry cycling reaction at 130 °C. The HRMS spectrum shows peaks for sodiated adducts of NOG and the peptide chain extension product containing the addition of a single alanine molecule on the NOG surface, which is indicated by NOG-Ala (cal: 433.3037; obs: 433.3040; mass error = 0.7 ppm).

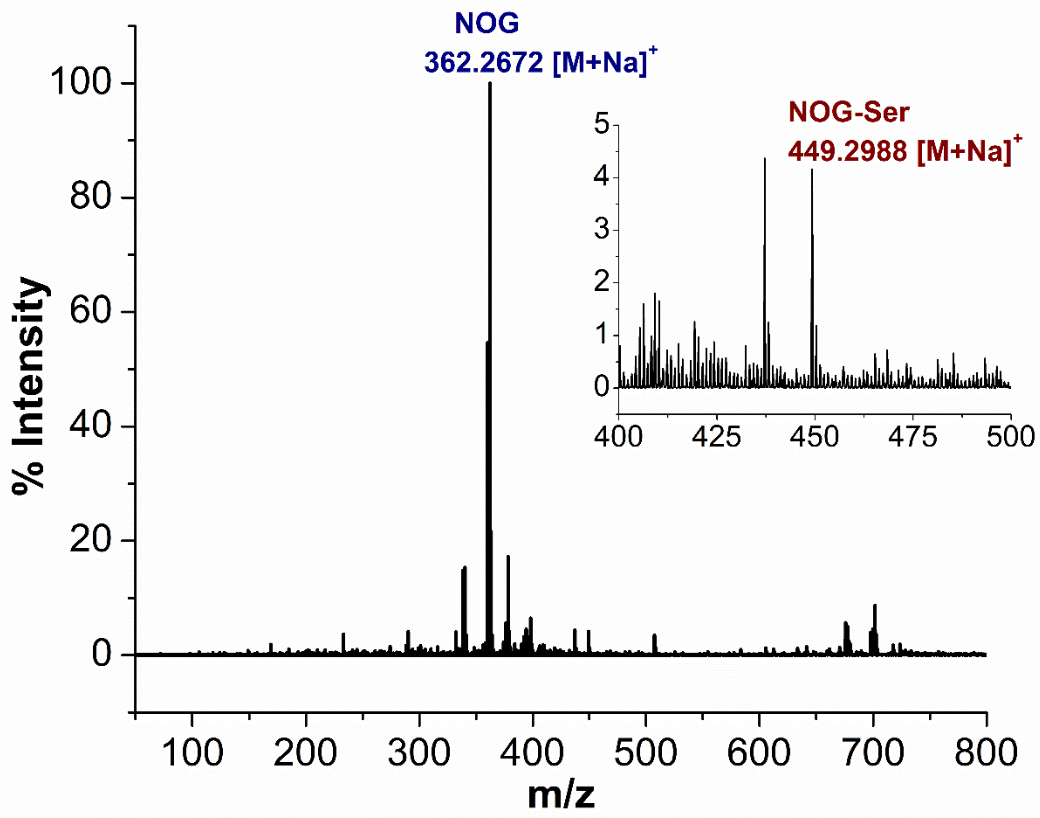

Supplementary Figure 26. HRMS analysis (positive mode) of the final lipid content of NOG + serine wet-dry cycling reaction at 130 °C. The HRMS spectrum shows peaks for sodiated adducts of NOG and the peptide chain extension product containing the addition of a single serine molecule on the NOG surface (see inset), which is indicated by NOG-Ser (cal: 449.2986; obs: 449.2988; mass error = 0.4 ppm).

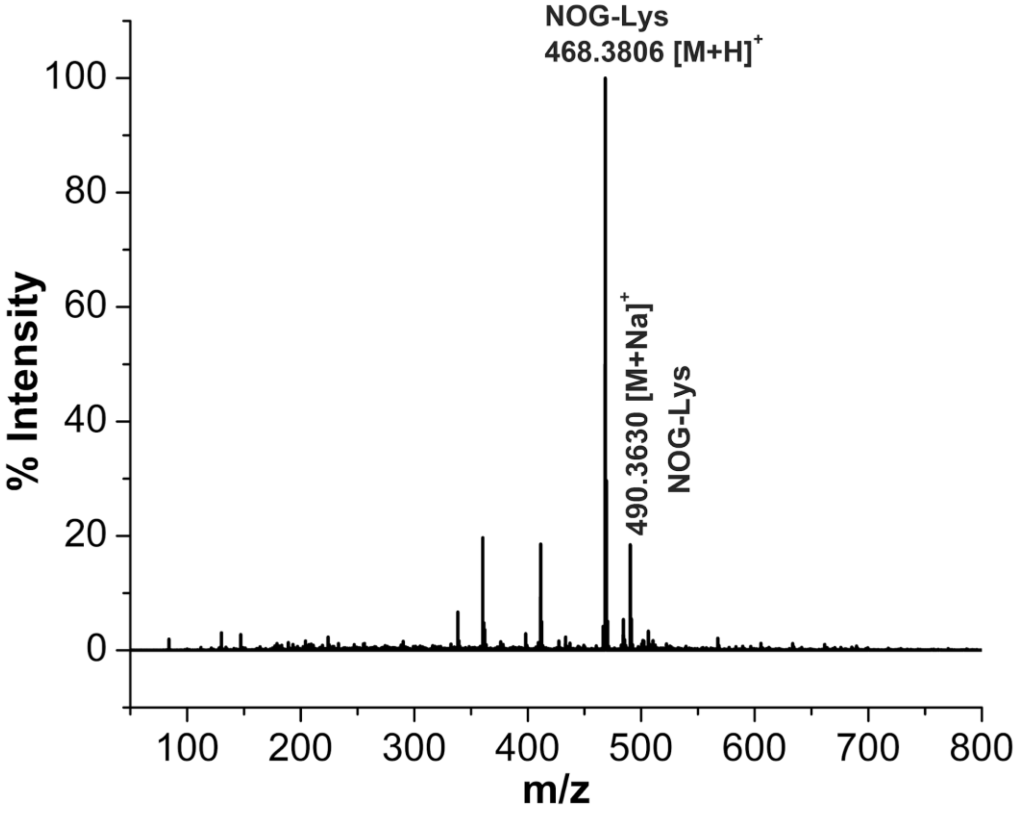

Supplementary Figure 27. HRMS analysis (positive mode) of the final lipid content of NOG + lysine wet-dry cycling reaction at 130 °C. The HRMS spectrum shows predominant peaks for the peptide chain extension product containing the addition of a single lysine molecule on the NOG surface, which is indicated by NOG-Lys. Unlike other amino acid extension products, for NOG-Lys, the most abundant peak was the protonated species ($[M+H]^+$; cal: 468.3796; obs: 468.3806; mass error = 2.1) followed by the sodiated adduct ($[M+Na]^+$; cal: 490.3615; obs: 490.3630; mass error = 3.1). Also, note that the peak for precursor NOG was not detected, which likely indicates the complete conversion of NOG to NOG-lys.
