# Supplementary material for: Elucidating N-acyl amino acids as a model protoamphiphilic system
Source: Commun Chem. 2022 Nov 9;5:147. doi: 10.1038/s42004-022-00762-9 (PMC9814278; doi:10.1038/s42004-022-00762-9)
Supplement: Supplementary file 1 — Rajamani_PR File [file 42004_2022_762_MOESM1_ESM.pdf]

nature portfolio

Peer Review File

Elucidating N-acyl amino acids as a model protoamphiphilic system

Open Access This file is licensed under a Creative Commons Attribution 4.0 International License, which permits use, sharing, adaptation, distribution and reproduction in any medium or format, as long as you give appropriate credit to the original author(s) and the source, provide a link to the Creative Commons license, and indicate if changes were made. In the cases where the authors are anonymous, such as is the case for the reports of anonymous peer reviewers, author attribution should be to 'Anonymous Referee' followed by a clear attribution to the source work. The images or other third party material in this file are included in the article's Creative Commons license, unless indicated otherwise in a credit line to the material. If material is not included in the article's Creative Commons license and your intended use is not permitted by statutory regulation or exceeds the permitted use, you will need to obtain permission directly from the copyright holder. To view a copy of this license, visit http://creativecommons.org/licenses/by/4.0/.

Reviewers' comments:

Reviewer #1 (Remarks to the Author):

The manuscript is well written, logically laid out, and easy to follow. The results are also interesting and of relevance to the origins of life field. Joshi et al. show that wet-dry cycles of monoolein and amino acid (usually Gly) leads to the formation of N-acyl amino acids, basically fatty acids connected to an amino acid through an amide bond. The resulting amphiphiles can form vesicles when mixed with either monoolein or oleate. Higher temperatures during the drying part of the wet-dry cycles leads to multiple additions of amino acids, which the authors suggest could have been important for the coupling of catalytically active peptides with protocells.

I would suggest that the authors move their TLC images from the main text to the supp section. They have nice mass spec data, so the TLC images in the main text seem redundant.

The microscopy images make use of an amphiphilic fluorophore (Octadecyl Rhodamine B Chloride) that partitions to the membrane. This should be mentioned in the figure legends of Figures 2 and 3.

Do the authors think that in Figures 2 and 3 that the membrane composition of the vesicles are unchanged across pH? If a vesicle contained an encapsulated, charged fluorophore, would that fluorophore be retained if the vesicle were moved to a solution of different pH?

Why is it more difficult to get multiple additions of amino acid when using Ala, Ser, and Lys as opposed to Gly? Do the authors see evidence of amino acid degradation at the elevated (130 °C) temperature that was exploited? There was a recent paper that showed that 150 °C leads to the degradation of amino acids during wet-dry cycling. Was the decreased addition of non-Gly amino acids due to such degradation?

The diagnostic peak in the proton NMR spectrum of Figure S4 is not very convincing. It looks like it could be noise. I do think that the authors have the molecule that they claim to have, since they have convincing mass spec data, but I'm not sure if this spectrum in Figure S4 is strengthening the case.

"Such membrane-anchored lipopeptides would have endowed early protocell membranes with new functions like catalytic activity" The word "would" here seems a bit too strong. "Could" makes more sense.

"In a prebiotic context, NAAs have been investigated for their ability to assist fatty acid-based membranes in vesicle growth and RNA localization on membrane surfaces16,17."
Perhaps the paper from the Saenz lab would also be relevant here:
https://www.pnas.org/doi/10.1073/pnas.2119235119

Reviewer #2 (Remarks to the Author):

In this manuscript, the authors report synthesis of N-acyl-amino acids from a novel reagent (monoglyceride), they investigate membrane assembly from mixtures of these N-acyl-amino acids together with other prebiotically plausible amphiphiles, and they show that N-acyl-amino acids can react with other single amino acids to form N-acyl peptides. The manuscript builds on previous work from the authors that demonstrated formation of these N-acyl-amino acids from ester-linked phospholipids, and demonstrated assembly of N-acyl-amino acids into membranes in the absence of other amphiphiles. This new manuscript is another solid contribution to their story, their conclusions are supported by the data, and their findings are of interest to the broader astrobiology community

because they illustrate that mixtures of amphiphiles on the early Earth can have surprising self-assembly behaviors. I support eventual publication of this manuscript, but I have a few questions and suggestions that need to be addressed first.

1) There seems to be a significant difference between the temperature at which N-acyl amino acids are synthesized (90°C) and the temperature where membranes have been observed (presumably about 20°C). Have the authors tried to determine the minimum temperature for synthesis of N-acyl amino acids? Have the authors tried to determine the upper temperature limit for membrane stability? If so, can the authors summarize these experiments in this paper? It would also be helpful if the authors could comment on possible early Earth environments where dry high-temperature regions (sites of amphiphile synthesis) could be in proximity to moderate temperature solutions (sites of membrane formation).

2) In the main text and Figure captions, it would be helpful if the authors were more explicit about their reaction conditions (time, temperature, pH). For example, the Fig 1 caption could say: "Conversion of GMO to NOG in the presence of Gly after three 24-hr wet-dry cycles at 90°C. The initial pH of the solution was 9.8." In Fig 4, the caption could say "The addition of amino acids to the NOG head group after 1 24-hr wet-dry cycle at 130°C results in the synthesis of N-acylated peptides. The initial pH of the solution was 9.8"

3) If GMO is the only amphiphile present, do vesicles form throughout pH range 4 – 11? This is important to understand the results in Fig 2. For example, if GMO alone forms vesicles throughout pH 4 – 11, how do we know that NOG is really even incorporated into the membrane? Figure 3 is beautiful because it provides a similar control with oleic acid, and demonstrates that mixtures of NOG + OA extend the vesicle stability regime to pH7. So I think that the authors should test whether GMO alone forms vesicles throughout pH range 4 – 11, and discuss these results in the context of Fig 2. The authors could instead cite papers from the literature if these experiments have been done previously.

4) The resolution of the microscope images is poor. For example in Fig 2, the pH 6 Fluorescence image is pixelated when I zoom in. I'm guessing the image shows a cluster of vesicles, but we need higher resolution images to be sure. I don't think that the authors need to take new images, but maybe they can save the image files in a different format to improve the resolution.

5) In the Fig 2 and Fig 3 captions, can the authors state which fluorescent dye is being imaged?

6) In the Figure 2 caption, and similar locations throughout the main text, I think it would be helpful to say "6 mM total amphiphile; 2:1 ratio" rather than just "6mM; 2:1 ratio".

7) I am surprised that OA alone forms vesicles at a very different range of pH values than NAAs alone (pH >8 vs pH <7). For both types of amphiphiles, the ionizable group is a carboxylic acid. Do you suspect that each carboxyl has a very different pKa in the bilayer? Please comment on this difference.

8) At the end of the main text, please put in an "Abbreviations" section with the chemical abbreviations that you use throughout (NOG, NAA, GMO, OA)

Reviewer #3 (Remarks to the Author):

The major claim of this paper is a convincing novel synthesis under prebiotic conditions of a class of membrane-forming amphiphiles whose head groups constitute monomeric and oligomeric (peptidic) amino acid derivatives. This is an important step towards elucidating the early appearance of potentially catalytic moieties on the surface of membrane-ensheathed protocells. These results also

significantly eliminate the prerequisite for the less likely emergence for of more complex amphiphiles, similar to lipids of present day cells, as described in a previous paper of these authors (manuscript, Ref 3). One more noteworthy achievement is adding evidence for the advantages of amphiphile admixtures in promoting the formation and maintenance of vesicular membranes under a broad range of environmental conditions.
In the conclusion the authors say: "Future research in this direction will appreciably contribute towards understanding how lipopeptides might have modulated physicochemical properties and the functionality of protocell membranes". I feel that this text does not sufficiently reveal crucial points that will be of interest to, and influence the thinking of others in the community and in the wider field. Two wide-open questions in the science of life's origin are how early protocells would acquire metabolism, and how protocells could undergo reproduction in their entirety. The present paper has a significant potential to shed light on such key items. I strongly suggest to augment the conclusion by pointing out the following: in-situ synthesis of catalytic amphiphile-attached peptides may constitute a step towards the role of membrane-residing catalysts in the emergence of metabolism [1], as exemplified by the scenario: "…gradually coevolving nucleic acids and proteins in interaction with increasingly complex cofactors, lipid membrane structures and other cellular components" [2]. Membrane-attached short peptide catalysts have also proposed to be essential for the inception of early protocellular reproduction [3], and in helping to solve the question of synchronizing membrane growth and the growth of molecular content [4]. In the same vein, the statement "…our study underscores the pivotal role played by NAAs in shaping the emergence and evolution of protocell membranes on primitive Earth" would truly benefit from explicitly pointing out that emerging of lipopeptides would not only improve vesicle environmental stability but also introduce stereospecificity, crucial for evolved life forms. It should be commented that in this respect, lipopeptides could serve as early enzyme-like catalysts that guide the formation of additional specific lipopeptides [5], mimicking the networks later becoming part of a metabolism.

Additional comments:
a) Please address the likelihood that under certain conditions a library of peptides with different sequences will form when more than one amino acid is used.
b) Could some sequences have better properties, hence proto-selection could kick in.
c) What is the prospect that in future exploration the energy supply for peptide bond formation would be chemical activation instead of temperature gradient, thus become more life-like?
d) Could certain reactions be better catalyzed by some of the sequences formed as per point 1.
e) Will help provide a definition of "protoamphiphiles", namely which amphiphiles belong to this class and which do not.
f) Fig. 1 is not explanatory regarding how OA is a "solubilizer" for another amphiphile. Is this a micellation effect? This should become explicit in text and figure.
g) The authors stress the importance of membrane formation at physiological pH7. Is this prebiotically relevant? In other words, are there indications that life arose and neutral pH?
h) Is there information on vesicle properties with NAAs involving alanine, lysine, serine, and valine?
i) Please attempt to provide explanation why non-Gly amino acids do not polymerize beyond adding on monomer?

References
[1] A. Wilson, M., Wei, C. & Pohorille, A. Towards Co-Evolution of Membrane Proteins and Metabolism. Orig Life Evol Biosph 44, 357–361 (2014). https://doi.org/10.1007/s11084-014-9393-2
[2] Caetano-Anollés, G.; Kim, K.M.; Caetano-Anollés, D. The phylogenomic roots of modern biochemist Origins of proteins, cofactors and protein biosynthesis. J. Mol. Evol. 2012, 74, 1–34.
[3] Kahana A. and Lancet, D. Self- reproducing catalytic micelles as nanoscopic protocell precursors, Nat. Rev. Chem., 2021, DOI: 10.1038/s41570-021-00329-7.
[4] Adamala K, Szostak JW. 2013 Competition between model protocells driven by an encapsulated catalyst. Nat. Chem. 5, 495–501. (doi:10.1038/nchem.1650)
[5] Sara Murillo-Sánchez, Damien Beaufils, Juan Manuel González Mañas, Robert Pascal, Kepa Ruiz-

Mirazo, Fatty acids' double role in the prebiotic formation of a hydrophobic dipeptide Chem Sci. 2016 May 1; 7(5): 3406–3413. doi: 10.1039/c5sc04796j

<u>Response to Reviewers' comments</u>

We would like to thank all the reviewers for their rigorous and in-depth analysis of our manuscript and highly appreciate their constructive feedback on our work. We firmly believe that, after incorporating the changes suggested by the reviewers, both the comprehensiveness and the clarity of our manuscript has now substantially improved. Below, we address point-by-point all the comments made by the reviewers, where we have mentioned the reviewer's comments in italics and our responses to those comments in blue.

Reviewer #1

The manuscript is well written, logically laid out, and easy to follow. The results are also interesting and of relevance to the origins of life field. Joshi et al. show that wet-dry cycles of monoolein and amino acid (usually Gly) leads to the formation of N-acyl amino acids, basically fatty acids connected to an amino acid through an amide bond. The resulting amphiphiles can form vesicles when mixed with either monoolein or oleate. Higher temperatures during the drying part of the wet-dry cycles leads to multiple additions of amino acids, which the authors suggest could have been important for the coupling of catalytically active peptides with protocells.

We thank the reviewer for appreciating the presentation style of our manuscript. We are also pleased to know that the reviewer found our work interesting and relevant for the Origin(s) of Life field.

- I would suggest that the authors move their TLC images from the main text to the supp section. They have nice mass spec data, so the TLC images in the main text seem redundant.

We thank the reviewer for their suggestion. Actually, we had pondered about whether to keep the TLC image in the main text or in the supplementary while preparing the figures for the manuscript. The TLC image in Figure 1b is a direct demonstration of the conversion of GMO to NOG, where the GMO spot present at the start of the reaction (lane 4) disappears completely, with the appearance of a new spot by the end of the reaction, which corresponds to NOG (lane 5). This result also nicely corroborates with the HRMS data shown in Fig 1c, where NOG and OA that appear in lane 5 of the TLC, were detected as predominant peaks in the HRMS spectrum. Given the above reasons, we decided to keep the TLC and the HRMS data together in the main text as a corroborating set of results, and would really prefer to keep it as is. We sincerely think this would be helpful for the readers to understand our basic result in a more straightforward manner.

- The microscopy images make use of an amphiphilic fluorophore (Octadecyl Rhodamine B Chloride) that partitions to the membrane. This should be mentioned in the figure legends of Figures 2 and 3.

We have now included the information about the amphiphilic fluorophore in the figure legends of Figures 2 and 3.

- Do the authors think that in Figures 2 and 3 that the membrane composition of the vesicles are unchanged across pH? If a vesicle contained an encapsulated, charged fluorophore, would that fluorophore be retained if the vesicle were moved to a solution of different pH?

We thank the reviewer for bringing up this interesting line of thought. The self-assembly behavior of mixed amphiphile systems is a very complex phenomenon. Although, the membrane composition of vesicles generated from these mixed amphiphiles is primarily governed by the starting amphiphile concentrations, several other factors also influence the membrane composition. For example, there is a dynamic equilibrium between the free amphiphile monomers and the ones that have been incorporated into the membrane. This equilibrium depends on the tendency of an amphiphile molecule to stay within the membrane, which in turn is dependent on its solubility, as well as the membrane's properties such as fluidity, polarity etc. All these parameters are likely to get affected upon changing reaction conditions like pH, temperature and the ionic strength of the medium. Furthermore, the equilibrium between free monomers and membrane-incorporated amphiphiles will also be influenced by the presence of other higher order structures (like micelles, droplets etc.) in the solution, along with the vesicles. The abundance of such higher order structures also depends on the above-mentioned reaction conditions. Therefore, subtle differences are bound to occur in the membrane composition of the vesicles across different pH values, although it will be hard to follow experimentally.

Also, the primary goal of the vesicle-based experiments in this paper was to check whether NOG containing mixed amphiphile systems are even able to self-assemble into vesicles over a diverse pH range. We are very much interested in pursuing the stability and physicochemical properties of the vesicles in our reaction, and understanding how these will change upon inducing changes in the environmental conditions such as pH, temperature etc. Particularly, we are invested in understanding the implications of the aforesaid on their permeability. However, the fluorescence intensity of a fluorophore is a pH-dependent property, which will vary upon changing the solution's pH. Standard permeability assays, which monitor change in fluorescence intensity to estimate the permeability, are mostly performed at a constant pH and hence do not encounter the above-mentioned problem. However, in our case, we will have to start with identifying a suitable fluorophore that can meet the requirements for performing such permeability assays at varying pH values. Given that all this would constitute an entire project in itself and will require a lot of initial standardization, it is beyond the scope of this short communication. Nonetheless, these are all very interesting set of experiments and we wish to assure the reviewer that we are invested in understanding this systematically in detail.

- Why is it more difficult to get multiple additions of amino acid when using Ala, Ser, and Lys as opposed to Gly? Do the authors see evidence of amino acid degradation at the elevated (130 °C) temperature that was exploited? There was a recent paper that showed that 150 °C leads to the degradation of amino acids during wet-dry cycling. Was the decreased addition of non-Gly amino acids due to such degradation?

We agree with the reviewer that amino acids do get degraded at elevated temperatures, which was reflected in the browning of the solution over the course of the reaction at 130 °C. Although it is possible that such thermal degradation rates might be different for different amino acids, this is unlikely to be the primary reason for the differential amino acid addition efficiencies of Gly versus other amino acids in our case. Thermal decomposition of amino acids are known to occur more drastically at 150 °C and higher temperatures [1] when compared to that at 130 °C that we have used in our reaction. Also, the starting

amino acid concentration was in far more excess (200 mM) than that of NOG (3 mM). Therefore, even if some degradation occurs at 130 °C, the amino acid would still likely be present in a surplus amount to react with the NOG.

Also, the addition of an amino acid on the NOG head group is basically a peptide bond formation reaction, and Gly is known to react much more efficiently as compared to other amino acids during abiotic peptide synthesis reactions. This phenomenon has been reported earlier for silica, alumina and clay-catalyzed peptide bond formation reactions, performed under wet-dry cycles or temperature fluctuations [2,3]. Lawless and Levi mention that the lower yield of Ala peptides as compared to that of Gly peptides is attributed to the higher activation energy of peptide bond formation in Ala than in Gly [3]. It is plausible that the increase in the complexity and the bulkiness of the side chains of amino acids (other than Gly), might reduce their efficiency to undergo abiotic oligomerization. In the revised manuscript, we have included a plausible explanation for the lower efficiency of amino acid addition on the NOG head group by amino acids other than Gly.

- The diagnostic peak in the proton NMR spectrum of Figure S4 is not very convincing. It looks like it could be noise. I do think that the authors have the molecule that they claim to have, since they have convincing mass spec data, but I'm not sure if this spectrum in Figure S4 is strengthening the case.

We do agree with the reviewer that the intensity of the signature peak (amide hydrogen of NOG) in ^{1}H-NMR of the reaction mixture is low. This is likely due to the exchange of the hydrogen atom of -NH with the NMR inactive proton present in the solvent molecule (-OH of methanol-d_4). However, we had to continue with the methanol-d_4 as a solvent because it was the only solvent in which all the lipid species of the reaction mixture were soluble. Nonetheless, we decided to report this data as it was showing the same signature peak that we had observed in the (more conclusive) NMR analysis of the NAA synthesis reaction that we reported in our previously study [4]. However, after considering the reviewer's comment, we have now given less emphasis on the NMR results in the main text of the revised manuscript.

- "Such membrane-anchored lipopeptides would have endowed early protocell membranes with new functions like catalytic activity" The word "would" here seems a bit too strong. "Could" makes more sense.

We have modified the sentence accordingly.

- "In a prebiotic context, NAAs have been investigated for their ability to assist fatty acid-based membranes in vesicle growth and RNA localization on membrane surfaces16,17."

Perhaps the paper from the Saenz lab would also be relevant here: https://www.pnas.org/doi/10.1073/pnas.2119235119

Although we appreciate reviewer's suggestion about including this reference, we humbly disagree with its relevance for the abovementioned sentence in the manuscript. This sentence essentially highlights the previous work done on N-acyl amino acids to demonstrate how they can be advantageous to fatty acid membranes. However, Saenz lab paper suggested by the reviewer talks about the ability of lipid membranes to modulate the activity of RNA through sequence-dependent interactions, where it uses membrane systems generated from phospholipids, fatty acids or their mixtures. As this paper is not related to N-acyl amino acids, or their role in facilitating fatty acid membranes, we would like to leave it out as a reference relevant to the abovementioned sentence in the manuscript.

Reviewer #2

In this manuscript, the authors report synthesis of N-acyl-amino acids from a novel reagent (monoglyceride), they investigate membrane assembly from mixtures of these N-acyl-amino acids together with other prebiotically plausible amphiphiles, and they show that N-acyl-amino acids can react with other single amino acids to form N-acyl peptides. The manuscript builds on previous work from the authors that demonstrated formation of these N-acyl-amino acids from ester-linked phospholipids, and demonstrated assembly of N-acyl-amino acids into membranes in the absence of other amphiphiles. This new manuscript is another solid contribution to their story, their conclusions are supported by the data, and their findings are of interest to the broader astrobiology community because they illustrate that mixtures of amphiphiles on the early Earth can have surprising self-assembly behaviors. I support eventual publication of this manuscript, but I have a few questions and suggestions that need to be addressed first.

We thank the reviewer for appreciating our work and supporting the publication of a revised version of our manuscript in *Communications chemistry*.

- There seems to be a significant difference between the temperature at which N-acyl amino acids are synthesized (90°C) and the temperature where membranes have been observed (presumably about 20°C). Have the authors tried to determine the minimum temperature for synthesis of N-acyl amino acids? Have the authors tried to determine the upper temperature limit for membrane stability? If so, can the authors summarize these experiments in this paper? It would also be helpful if the authors could comment on possible early Earth environments where dry high-temperature regions (sites of amphiphile synthesis) could be in proximity to moderate temperature solutions (sites of membrane formation).

In this work, we have mostly used the previously standardized conditions for N-acyl amino acid (NAA) synthesis and their vesicle formation, which have been reported in our earlier studies [4,5]. While standardizing these conditions, especially for the NAA synthesis part, we also considered the previous information available on the ester-amide bond exchange process via which NAA formation occurs. The ester-amide exchange reaction has been extensively studied in the context of depsipeptide formation from a mixture of hydroxy acids and amino acids [6]. In this work, authors have also discerned the minimum temperature required for the ester-amide bond exchange, where they report that this process can occur even at temperatures like 65 °C.

We would also like to highlight that although the microscopy of vesicles has been performed at a lower temperature, the vesicle formation from NOG actually requires higher temperatures like 60 °C. Furthermore, this higher temperature also needs to be maintained to keep NOG in a vesicle form (we have explained in detail the protocol for NOG vesicle formation in the methods section of our manuscript). Therefore, there is not a significant difference between the temperatures at which NAAs are synthesized and the temperatures at which their membranes form and are sustained. In fact, this temperature stability of NAA-based vesicles could provide them a selective advantage over fatty acids to generate protocell membranes in high temperature geochemical settings like hot springs that have been proposed as a potential niche for life's origin. Notably, such hot springs can also serve as a site for NAA synthesis via their pertinent feature of wet-dry cycles.

As far as relevant early Earth environments are concerned, different terrestrial hot springs can have different temperatures. Furthermore, the same hot spring can also possess variable temperature niches, where the origin site has higher temperature than that of the run off site. Also, intermediate temperature

settings can be generated by the mixing of water from two source pools with different temperatures. All these plausible scenarios of terrestrial hot springs, could have met the temperature-specific requirements for various prebiotic reactions and self-assembly processes that occurred on the early Earth. In view of the reviewer's comment, we have now discussed in the revised manuscript the temperature requirements for NAA synthesis and their membrane formation, while also commenting on the plausible early Earth environments that could have met these requirements.

- In the main text and Figure captions, it would be helpful if the authors were more explicit about their reaction conditions (time, temperature, pH). For example, the Fig 1 caption could say: "Conversion of GMO to NOG in the presence of Gly after three 24-hr wet-dry cycles at 90°C. The initial pH of the solution was 9.8." In Fig 4, the caption could say "The addition of amino acids to the NOG head group after 1 24-hr wet-dry cycle at 130°C results in the synthesis of N-acylated peptides. The initial pH of the solution was 9.8"

We thank the reviewer for this suggestion; we have modified the main text and figure legends accordingly.

- If GMO is the only amphiphile present, do vesicles form throughout pH range 4 – 11? This is important to understand the results in Fig 2. For example, if GMO alone forms vesicles throughout pH 4 – 11, how do we know that NOG is really even incorporated into the membrane? Figure 3 is beautiful because it provides a similar control with oleic acid, and demonstrates that mixtures of NOG + OA extend the vesicle stability regime to pH7. So I think that the authors should test whether GMO alone forms vesicles throughout pH range 4 – 11, and discuss these results in the context of Fig 2. The authors could instead cite papers from the literature if these experiments have been done previously.

It is well known that GMO does not form vesicles on its own. It can do so only in the presence of additives like other lipids, detergents or salts [7]. The critical packing parameter (γ) of GMO molecule is 3.8, which does not allow it to spontaneously assemble into spherical structures like vesicles that can be generated only when the γ value of an amphiphile is in the range of 0 to 1/3 [7]. Also, GMO has very low solubility in water. Therefore, it does not go into the aqueous phase when dry lipid film of GMO is hydrated with water. This was the primary reason why we used a mixed system of OA + GMO in the NAA synthesis reaction, where the addition of OA increased the solubility of GMO via vesicle formation.

As suggested by the reviewer, we performed a control experiment to check whether GMO can form vesicles at all, and also to confirm the increased solubility of GMO upon OA addition. The results of this has been reported in the Figure S2 of the revised supplementary information. In this experiment, we hydrated lipid films containing only GMO and GMO + OA with 200 mM Gly pH 9.8 solution. The TLC analysis of the resultant solutions showed that GMO gets solubilized in water in the presence OA (Lane 3), but not on its own (Lane 1). Furthermore, the microscopic analysis of the solution containing OA + GMO showed the presence of vesicles. However, we did not observe any vesicles in the case of only GMO solution. Although GMO does not form vesicles by itself, it allows fatty acids to generate vesicles towards the more alkaline pH regimes from their usual vesicle formation pH range [8]. Therefore, in this study we explored whether a similar phenomenon is observed for NOG + GMO mixed system as well.

- The resolution of the microscope images is poor. For example in Fig 2, the pH 6 Fluorescence image is pixelated when I zoom in. I'm guessing the image shows a cluster of vesicles, but we need higher resolution images to be sure. I don't think that the authors need to take new images, but maybe they can save the image files in a different format to improve the resolution.

We apologise for the problem faced by the reviewer while observing the microscopy data when zoomed in. Actually, the images that we have prepared for the main paper and the supplementary figures are of high resolution (600 dpi). However, we suspect that the image quality might have decreased while converting manuscript into a PDF format. Nonetheless, we have separately provided high resolution images of the main paper figures along with the revised manuscript, which should hopefully resolve this problem.

- In the Fig 2 and Fig 3 captions, can the authors state which fluorescent dye is being imaged?

The fluorescent dye used for staining vesicles was Octadecyl Rhodamine B Chloride (R18). We have now included this information in the captions of Figures 2 and 3.

- In the Figure 2 caption, and similar locations throughout the main text, I think it would be helpful to say "6 mM total amphiphile; 2:1 ratio" rather than just "6mM; 2:1 ratio".

We have made the suggested changes in the main text and figure legends.

- I am surprised that OA alone forms vesicles at a very different range of pH values than NAAs alone (pH >8 vs pH <7). For both types of amphiphiles, the ionizable group is a carboxylic acid. Do you suspect that each carboxyl has a very different pKa in the bilayer? Please comment on this difference.

Although both fatty acids and NAAs have a carboxyl moiety at the terminal position of their head group, we suspect that the presence of an amide linkage in NAAs potentially lowers the apparent pKa of their head group, thereby allowing them to form vesicles at lower pH values. We have discussed this possibility in our previous paper [4], where we reported the vesicle formation behavior of NAAs for the first time.

- At the end of the main text, please put in an "Abbreviations" section with the chemical abbreviations that you use throughout (NOG, NAA, GMO, OA)

Although we have provided full forms of these abbreviations at the time of their first use in the main text, we will be happy to include a separate "Abbreviations" section if the journal wants and allows us to do so.

Reviewer #3

The major claim of this paper is a convincing novel synthesis under prebiotic conditions of a class of membrane-forming amphiphiles whose head groups constitute monomeric and oligomeric (peptidic) amino acid derivatives. This is an important step towards elucidating the early appearance of potentially catalytic moieties on the surface of membrane-ensheathed protocells. These results also significantly eliminate the prerequisite for the less likely emergence for of more complex amphiphiles, similar to lipids of present day cells, as described in a previous paper of these authors (manuscript, Ref 3). One more noteworthy achievement is adding evidence for the advantages of amphiphile admixtures in promoting the formation and maintenance of vesicular membranes under a broad range of environmental conditions.

We thank the reviewer for highlighting and appreciating the novelty and significance of our work.

- In the conclusion the authors say: "Future research in this direction will appreciably contribute towards understanding how lipopeptides might have modulated physicochemical properties and the functionality of protocell membranes". I feel that this text does not sufficiently reveal crucial points that will be of interest to, and influence the thinking of others in the community and in the wider field. Two wide-open questions in the science of life's origin are how early protocells would acquire metabolism, and how

protocells could undergo reproduction in their entirety. The present paper has a significant potential to shed light on such key items. I strongly suggest to augment the conclusion by pointing out the following: in-situ synthesis of catalytic amphiphile-attached peptides may constitute a step towards the role of membrane-residing catalysts in the emergence of metabolism [1], as exemplified by the scenario: "…gradually coevolving nucleic acids and proteins in interaction with increasingly complex cofactors, lipid membrane structures and other cellular components" [2]. Membrane-attached short peptide catalysts have also proposed to be essential for the inception of early protocellular reproduction [3], and in helping to solve the question of synchronizing membrane growth and the growth of molecular content [4]. In the same vein, the statement "…our study underscores the pivotal role played by NAAs in shaping the emergence and evolution of protocell membranes on primitive Earth" would truly benefit from explicitly pointing out that emerging of lipopeptides would not only improve vesicle environmental stability but also introduce stereospecificity, crucial for evolved life forms. It should be commented that in this respect, lipopeptides could serve as early enzyme-like catalysts that guide the formation of additional specific lipopeptides [5], mimicking the networks later becoming part of a metabolism.

We really appreciate and acknowledge the reviewer's insightful approach on the major open questions in the origin of life field, and how our work can provide a step towards addressing these questions. We also thank the reviewer for providing constructive feedback to improve the comprehensiveness of our discussion and conclusions part. The main reason that we did not overemphasize the catalytic role of lipopeptides towards developing a protometabolic network inside protocells, and also their potential role in improving protocell stability is because we have not performed any experiments in this context in this study,. However, we do very much agree with the reviewer that these are interesting future research directions that should be pursued. Therefore, to stimulate readers' thinking in these directions, we have revised the conclusion part of our manuscript by adding the following paragraphs:

"Two major open questions in the origin of life research pertain to how early protocells would acquire metabolism, and how would they have undergone reproduction in their entirety. The in-situ synthesis of catalytic lipopeptides may constitute a step towards understanding the role of membrane-residing catalysts in the emergence of early metabolism[30]. Notably, membrane adherence of short peptide catalysts, and the peptide products of such reactions have also been proposed to be useful for the onset of early protocellular reproduction[31], and synchronizing growth of membrane and its molecular content[32]."

"It will be interesting to further explore how such lipoamino acids and lipopeptides could have contributed towards increasing protocellular stability, the emergence of stereoselectivity, and the onset of early metabolism via their catalytic activity."

Additional comments:

- Please address the likelihood that under certain conditions a library of peptides with different sequences will form when more than one amino acid is used.

- Could some sequences have better properties, hence proto-selection could kick in.

- Could certain reactions be better catalyzed by some of the sequences formed as per point 1.

We have included a short paragraph in the lipopeptide synthesis section to address the likelihood of the abovementioned scenarios, which reads as follows:

“This diversity can be further increased in terms of the peptide sequence of these lipopeptides by using more than one amino acids in the starting reaction mixture. Increasing their sequence diversity might allow certain lipopeptides to acquire new advantageous properties like catalytic activity, which could provide them a selective advantage over other lipopeptides and for e.g. allow to become a part of emerging protocells.”

- What is the prospect that in future exploration the energy supply for peptide bond formation would be chemical activation instead of temperature gradient, thus become more life-like?

It is possible that at some stage during the evolution of life, the energy source required to drive the thermodynamically unfavorable synthesis of biologically-relevant polymers would have been shifted from environmental conditions like temperature or pH gradients to chemical activation. This would be applicable for peptide bond formation as well. In our previous paper where we looked at the effect of a membrane-forming amphiphile on abiotic peptide synthesis, we have discussed both the above-mentioned modes of peptide synthesis [4]. The synthesis of lipoamino acids and lipopeptides, which involves peptide bond formation, can also be achieved by utilizing energy from environmental sources like temperature (the approach used in this study), as well as via activation chemistry [9,10]. We have included this point in the abovementioned context in the NAA synthesis section of the manuscript.

- Will help provide a definition of "protoamphiphiles", namely which amphiphiles belong to this class and which do not.

As defined in the abstract and the introduction sections of our manuscript, protoamphiphiles are those amphiphiles which were likely present on the prebiotic Earth and potentially served as membrane components of early protocells. This is a new terminology that we first introduced in our previous paper on N-acyl amino acids [4]. Similar words for other molecules/assemblies/networks in the origin of life field include protocells, protopeptides, protoRNA, protometabolism etc. However, we noticed that there was no such parallel term for amphiphiles in a prebiotic context. Hence, we decided to introduce the term “protoamphiphiles”, and are now using this term in our scientific write-ups. Since it is a new terminology, we have kept it broad in terms of its definition to encompass a large array of currently studied (and yet to be discovered) prebiotically plausible amphiphiles, which are of varied chain lengths and possess a variety of head group types. So, by definition, any prebiotically plausible amphiphile that would have potentially been part of protocell membranes, could be considered as a “protoamphiphile”.

- Fig. 1 is not explanatory regarding how OA is a "solubilizer" for another amphiphile. Is this a micellation effect? This should become explicit in text and figure.

GMO itself is poorly soluble in water. OA increases its solubility through the formation of vesicles which can incorporate the GMO molecules into them, thereby allowing them to mix with the aqueous phase. This strategy of increasing the water solubility of monoglycerides by mixing them with fatty acids, which results in the blended vesicles of fatty acids and monoglycerides in the aqueous solution, has been used previously [11]. To validate this overall phenomenon, we performed a control experiment, the result of which has been reported in the Figure S2 of the revised supplementary information. In this experiment,

we hydrated lipid films containing only GMO, and GMO + OA, with 200 mM Gly pH 9.8 solution. The TLC analysis of the resultant solutions showed that GMO gets solubilized in water in the presence of OA (Lane 3), but not on its own (Lane 1). Furthermore, the microscopic analysis of the solution containing OA + GMO showed the presence of vesicles. However, we did not observe any vesicles in the case of the only GMO solution. This experiment confirms that OA increases the aqueous solubility of GMO via vesicle formation.

- The authors stress the importance of membrane formation at physiological pH7. Is this prebiotically relevant? In other words, are there indications that life arose and neutral pH?

N-acyl amino acids perform a variety of functions in biological cells [12], and they also have commercial applications in developing efficient drug delivery systems [13], both of which operate at a physiological pH. When we emphasized the vesicle formation by NAA-based amphiphiles at a physiological pH, we intended to highlight the implication of this result in the abovementioned context and not from the origin of life point of view. We apologise for not communicating it explicitly. We have now modified the main text accordingly to make this point clear.

- Is there information on vesicle properties with NAAs involving alanine, lysine, serine, and valine?

Apart from Gly containing NAAs, the only other commercially available NAA is N-oleoyl serine (NOS), whose vesicle formation behavior has been reported in our previous study [4]. Notably, NOS forms vesicles in the pH regime of 3 to 5, which is more acidic as compared to the vesicle formation range of NOG (pH 5 to 6). We postulate that this decrease in the pH optimum for NOS vesicle formation is because of the presence of an electron withdrawing hydroxyl group in the side chain of serine, which could potentially make the terminal carboxyl group of NOS more acidic compared to that of NOG.

There is no information available on the vesicle formation behavior of NAAs containing other amino acid head groups like alanine, lysine, valine etc. We are actively working towards chemically synthesizing these other NAAs in the lab and studying their physicochemical properties and self-assembly behavior.

- Please attempt to provide explanation why non-Gly amino acids do not polymerize beyond adding on monomer?

The addition of an amino acid on the NOG head group is basically a peptide bond formation reaction, and Gly is known to react much more efficiently as compared to other amino acids during abiotic peptide synthesis reactions. This phenomenon has been reported earlier for silica, alumina and clay-catalyzed peptide bond formation reactions performed under wet-dry cycles or temperature fluctuations [2,3]. Lawless and Levi mention that the lower yield of Ala peptides as compared to that of Gly peptides is attributed to the higher activation energy of peptide bond formation for Ala than for Gly [3]. It is plausible that the increase in the complexity and the bulkiness of side chains of amino acids other than Gly might reduce their efficiency to undergo abiotic oligomerization. In the revised manuscript, we have included a plausible explanation for the lower efficiency of amino acid addition on the NOG head group by amino acids other than Gly.

References

1. Rodriguez-Garcia, M.; Surman, A.J.; Cooper, G.J.T.; Suárez-Marina, I.; Hosni, Z.; Lee, M.P.; Cronin, L. Formation of oligopeptides in high yield under simple programmable conditions. *Nat. Commun.* **2015**, *6*, 8385.

2. Bujdák, J.; Rode, B.M. Silica, Alumina and Clay Catalyzed Peptide Bond Formation: Enhanced Efficiency of Alumina Catalyst. *Orig. life Evol. Biosph. 1999 295* **1999**, *29*, 451–461.

3. Lawless, J.G.; Levi, N. The role of metal ions in chemical evolution: Polymerization of alanine and glycine in a cation-exchanged clay environment. *J. Mol. Evol. 1979 134* **1979**, *13*, 281–286.

4. Joshi, M.P.; Sawant, A.A.; Rajamani, S. Spontaneous emergence of membrane-forming protoamphiphiles from a lipid–amino acid mixture under wet–dry cycles. *Chem. Sci.* **2021**, *12*, 2970–2978.

5. Joshi, M.P.; Steller, L.; Van Kranendonk, M.J.; Rajamani, S. Influence of Metal Ions on Model Protoamphiphilic Vesicular Systems: Insights from Laboratory and Analogue Studies. *Life 2021, Vol. 11, Page 1413* **2021**, *11*, 1413.

6. Forsythe, J.G.; Yu, S.-S.; Mamajanov, I.; Grover, M.A.; Krishnamurthy, R.; Fernández, F.M.; Hud, N. V. Ester-Mediated Amide Bond Formation Driven by Wet-Dry Cycles: A Possible Path to Polypeptides on the Prebiotic Earth. *Angew. Chemie* **2015**, *127*, 10009–10013.

7. Kulkarni, C. V.; Wachter, W.; Iglesias-Salto, G.; Engelskirchen, S.; Ahualli, S. Monoolein: a magic lipid? *Phys. Chem. Chem. Phys.* **2011**, *13*, 3004–3021.

8. Sarkar, S.; Dagar, S.; Verma, A.; Rajamani, S. Compositional heterogeneity confers selective advantage to model protocellular membranes during the origins of cellular life. *Sci. Rep.* **2020**, *10*, 1–11.

9. Izgu, E.C.; Björkbom, A.; Kamat, N.P.; Lelyveld, V.S.; Zhang, W.; Jia, T.Z.; Szostak, J.W. N-Carboxyanhydride-Mediated Fatty Acylation of Amino Acids and Peptides for Functionalization of Protocell Membranes. *J. Am. Chem. Soc.* **2016**, *138*, 16669–16676.

10. Bonfio, C.; Russell, D.A.; Green, N.J.; Mariani, A.; Sutherland, J.D. Activation chemistry drives the emergence of functionalised protocells. *Chem. Sci.* **2020**, *11*, 10688–10697.

11. Novales, B.; Ropers, M.H.; Douliez, J.P. Use of fatty acid/monoglyceride vesicle dispersions for stabilizing O/W emulsions. *Colloids Surfaces A Physicochem. Eng. Asp.* **2005**, *269*, 80–86.

12. Battista, N.; Bari, M.; Bisogno, T. N-Acyl Amino Acids: Metabolism, Molecular Targets, and Role in Biological Processes. *Biomol. 2019, Vol. 9, Page 822* **2019**, *9*, 822.

13. M. Ziora, Z.; A. Blaskovich, M.; Toth, I.; A. Cooper, M. Lipoamino Acids as Major Components of Absorption Promoters in Drug Delivery. *Curr. Top. Med. Chem.* **2012**, *12*, 1562–1580.

REVIEWERS' COMMENTS:

Reviewer #1 (Remarks to the Author):

I am satisfied with the responses to my comments and look forward to seeing this manuscript in print.

Reviewer #2 (Remarks to the Author):

The authors have addressed my comments. I support publication of their article.

Reviewer #3 (Remarks to the Author):

Editorial note: this reviewer provided no further comments for the authors
